# Voltage-sensor movements in the Eag Kv channel under an applied electric field

**DOI:** 10.1073/pnas.2214151119

**Published:** 2022-11-07

**Authors:** Venkata Shiva Mandala, Roderick MacKinnon

**Affiliations:** ^a^Laboratory of Molecular Neurobiology and Biophysics, The Rockefeller University, New York, NY, 10065;; ^b^HHMI, The Rockefeller University, New York, NY, 10065

**Keywords:** Eag channel, voltage sensor, membrane potential, cryo-EM, potassium channel

## Abstract

Voltage-dependent ion channels regulate the opening of their pores by sensing the membrane voltage. This process underlies the propagation of action potentials and other forms of electrical activity in cells. The voltage dependence of these channels is governed by the transmembrane displacement of the positive charged S4 helix within their voltage-sensor domains. We use cryo-electron microscopy to visualize this movement in the mammalian Eag voltage-dependent potassium channel in lipid membrane vesicles with a voltage difference across the membrane. Multiple structural configurations show that the applied electric field displaces S4 toward the cytoplasm by two helical turns, resulting in an extended interfacial helix near the inner membrane leaflet. The position of S4 in this down conformation is sterically incompatible with an open pore, thus explaining how movement of the voltage sensor at hyperpolarizing membrane voltages locks the pore shut in this kind of voltage-dependent K^+^ (K_v_) channel. The structures solved in lipid bilayer vesicles detail the intricate interplay between K_v_ channels and membranes, from showing how arginines are stabilized deep within the membrane and near phospholipid headgroups, to demonstrating how the channel reshapes the inner leaflet of the membrane itself.

## Significance

Voltage-dependent ion channels underlie the action potential and other forms of electrical activity in cells. They have been the subjects of much study since Hodgkin and Huxley described the electrical origins of the action potential in 1952. Over the past two decades, structures of voltage-dependent K^+^, Na^+^, Ca^2+^, hyperpolarization-activated cyclic nucleotide–gated and transient receptor potential channels have been determined. The biggest question that remains regarding the structure and mechanism of this entire class of ion channels is: How do the voltage sensors respond to an applied electric field across the membrane? This study presents structures of the Eag voltage-dependent K^+^ channel in electrically polarized lipid vesicles, using cryo-electron microscopy and showing how the voltage sensors regulate the pore.

In voltage-dependent ion channels, the transmembrane voltage determines whether the pore opens. At the same time, the flow of ions through the open pore alters the membrane voltage by charging the membrane capacitance. This recursive regulation of ion channel activity by membrane voltage is the fundamental process at the heart of cellular electricity. As described by Hodgkin and Huxley (1), voltage-dependent membrane permeability to Na^+^ and K^+^ (ion channels as molecular entities had not yet been discovered) generates the action potential, which is by far the most rapid form of information transfer across long distances in cells. Voltage-dependent ion channels underlie many other aspects of cell signaling as well, including the initiation of muscle contraction by voltage-dependent Ca^2+^ channels (2, 3) and the control of cardiac and neuronal pacemaker frequency by the hyperpolarization-activated cyclic nucleotide–gated (HCN) channel (4, 5).

Voltage-dependent ion channels contain structural domains called voltage sensors that control pore opening by membrane voltage. Whether in K^+^, Na^+^, Ca^2+^, or cation channels like HCN channels or transient receptor potential (TRP) channels, voltage sensors have a conserved structure comprising four transmembrane helices, named S1, S2, S3, and S4 (3, 6, 7). The fourth helix, S4, contains repeats of the amino acid triplet (RXX)_n_, where R stands for arginine, sometimes substituted by lysine, X for hydrophobic amino acid, and n varies widely among different channels. At the center of voltage sensors, inside the membrane’s interior, a constellation of negative charged amino acids, aspartate or glutamate, and a phenylalanine residue forms a gating-charge transfer center that stabilizes the positive charged side chains of arginine and lysine as they cross the membrane (8, 9). In some voltage sensors, S4 undergoes a transition from an α to a 3_10_ helix to direct the arginine and lysine side chains of S4 into the gating-charge transfer center (8–11). The displacement of S4 across the membrane is detectable as a nonlinear capacitive current, called gating current in electrophysiology experiments (9, 12), and is ultimately responsible for voltage control of a voltage-dependent ion channel’s pore.

So far, voltage-dependent ion channel structures have been determined in crystals, detergent micelles, or nanodiscs without a voltage difference across them (8, 13–19). Under such conditions, most voltage sensors adopt a depolarized conformation, which is expected in a membrane at 0 mV. Chemical cross links, metal affinity bridges, mutations, and toxins have been used to capture or stabilize voltage sensors in conformations thought to mimic the hyperpolarized (i.e., negative voltage inside) condition (10, 11, 20–23). Here, we present a cryo-electron microscopy (cryo-EM) analysis of the mammalian Eag voltage-dependent K^+^ (K_v_) channel in lipid membrane vesicles with a voltage generated across the membrane using K^+^ ion gradients in the presence of valinomycin. Doing so allows us to not only visualize how the voltage sensors respond to the applied electric field but also to see how the lipid membrane near the channel, which is intimately tied to the function of voltage-dependent ion channels, is reshaped by these conformational changes.

## Results

### Rationale for Polarizing Eag Channels.

The Eag K^+^ channel (24–26) was chosen for study because its pore is gated by both membrane voltage and intracellular Ca^2+^ through separate molecular mechanisms. Four voltage sensors inside the membrane regulate the gate in response to membrane voltage, while four calmodulin subunits in the cytoplasm regulate the gate by closing it in the presence of Ca^2+^ (18). From electrophysiology studies, in the absence of regulation by calmodulin, one would expect Eag to exhibit depolarized voltage sensors and an open pore at 0 mV (27, 28). Indeed, this is precisely what we observe in the structure of the hERG K^+^ channel, which is very similar to Eag in sequence and structure but lacks calmodulin regulation (17). Assuming that Eag behaves like hERG, the advantage to studying voltage sensor movements in Eag, with its pore held closed by Ca^2+^ and calmodulin, is that its voltage sensors can be driven into their hyperpolarized conformation without performing additional work to close the pore. An applied electric field has only to overcome the energy difference between depolarized and hyperpolarized voltage sensors.

### Eag Reconstitution and Polarization.

**Fig. 1.**
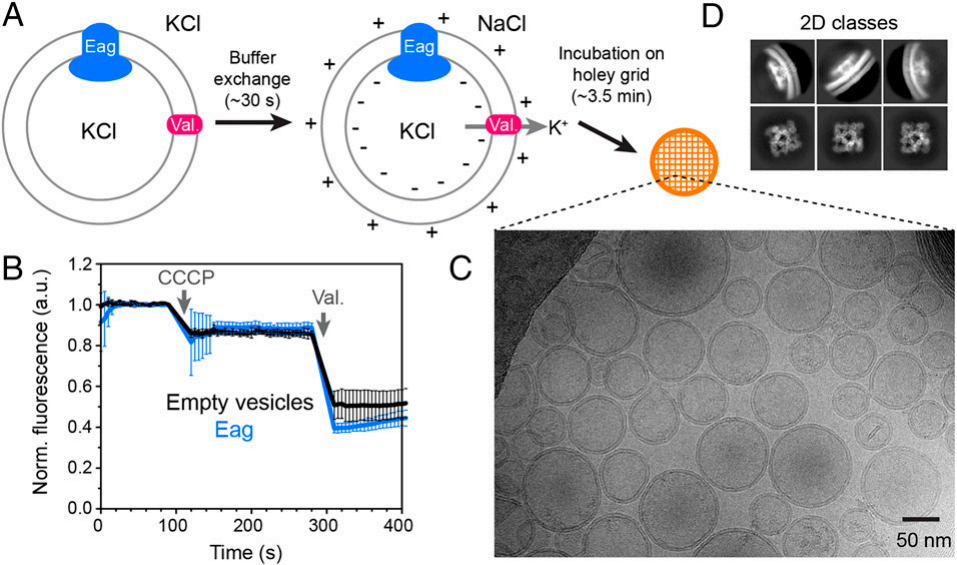
Preparation of polarized Eag-containing liposomes. (*A*) Schematic of protocol used to obtain polarized vesicles for cryo-EM analysis. Eag is reconstituted into liposomes with symmetrical KCl and valinomycin is added to mediate potassium flux. External KCl is exchanged for NaCl using a buffer-exchange column. Potassium efflux through valinomycin generates a potential difference across the membrane such that the inside of the vesicle is negative with respect to the outside. Vesicles are frozen on a holey carbon grid for structure determination. (*B*) Liposome-based flux assay to test polarization of vesicles. Recordings (*n* = 3, mean ± SD) were made using empty vesicles (black) or vesicles with Eag (blue). Addition of the proton ionophore CCCP allows proton entry, detected by quenching a fluorophore (ACMA). Protons enter when a K^+^ ionophore, valinomycin, is added. (*C*) Representative micrograph showing vesicles (scale bar, 50 nm) and (*D*) 2D class averages of the membrane-embedded channel. a.u., arbitrary units; Norm., normalized; Val., valinomycin.

The rat ortholog of Eag1 (termed Eag) (18) was purified with Ca^2+^ and calmodulin (*SI Appendix*, Fig. S1 *A* and *B*) and reconstituted into a ratio of 90:5:5 1-palmitoyl-2-oleoyl-*sn*-glycero-3-phosphocholine (POPC) to 1-palmitoyl-2- oleoyl-*sn*-glycero-3-phosphoglycerol (POPG) to cholesterol [wt/wt/wt] liposomes with 300 mM KCl. For the cryo-EM samples, valinomycin was added and the external solution was exchanged to 300 mM NaCl using a buffer-exchange column. The potassium gradient results in valinomycin-mediated K^+^ efflux from the vesicles and generation of a voltage across the membrane (Fig. 1*A*). The K^+^ concentrations inside (300 mM) and outside (measured to be ∼1 mM) the vesicles give an upper limit for the voltage difference of approximately −145 mV. These polarized vesicles were immediately applied to a holey carbon grid and frozen (Fig. 1*C*). To test whether Eag-containing liposomes can maintain a voltage difference across the membrane on the timescale of grid freezing, we used a liposome flux assay (Fig. 1*B*) (29). Vesicles reconstituted in 300 mM KCl were diluted into a buffer containing isotonic NaCl (to generate a K^+^ gradient) and a fluorescent dye, 9-amino-6-chloro-2-methoxyacridine (ACMA). The H^+^ ionophore carbonyl cyanide *m*-chlorophenylhydrazone (CCCP) was then added to allow membrane voltage–driven proton flux into the vesicle, which, in turn, results in quenched ACMA fluorescence due to protonation of the dye. Upon addition of CCCP, no flux was detected in vesicles with or without Eag, consistent with the channel being tightly closed by calmodulin under these conditions (Fig. 1*B*). Addition of the K^+^ ionophore valinomycin led to rapid quenching of ACMA in both sets of vesicles, indicating that valinomycin-mediated K^+^ efflux generates a membrane potential in Eag vesicles that is stable for at least a few minutes. In summary, we made polarized vesicles containing Eag such that the voltage inside is negative with respect to the outside.

### Identification of Three Structural Classes.

We determined the structures of Eag in the polarized vesicles using single-particle cryo-EM analysis (*SI Appendix*, Figs. S1*C* and S2 and Table S1). Representative micrographs showed mostly unilamellar vesicles with a diameter of 30 to 100 nm (Fig. 1*C*). One might expect to see channels reconstituted in two orientations, either inside-in or inside-out. However, side views in two-dimensional (2D) classes only showed channels in the inside-in orientation (i.e., inserted such that their cytoplasmic domains (CTDs) face the vesicle interior) (Fig. 1*D* and *SI Appendix*, Fig. S1*C*). All channels are thus oriented as they would be in cells and experience hyperpolarizing conditions under the applied electric field. Both 2D and three-dimensional (3D) classifications were first used to identify a good subset of particles. To isolate movements in the voltage sensor, we performed focused refinement on the pore domain and CTD while excluding the voltage-sensor domain. Subsequent classification of the transmembrane (TM) domain showed two classes, with roughly equal particle counts, that had both well-resolved pore domains as well as visible voltage-sensor density (*SI Appendix*, Fig. S2). This procedure was repeated using an independent reconstruction (for details, see *SI Appendix*, Fig. S2, and *Materials and Methods*), and further classification of the two populations, either imposing C4 symmetry or without symmetry on a symmetry-expanded particle set, yielded three interpretable cryo-EM density maps referred to hereafter as “up,” “intermediate,” and “down.” The up map (C4 symmetric) comes from the class with better-defined voltage sensor density and had an overall resolution of 3.9 Å (*SI Appendix*, Fig. S3). The intermediate and down maps (no symmetry) arise from the class with a more heterogeneous voltage sensor and have overall resolutions of 4.9 Å and 5.4 Å, respectively. The rationale for the map nomenclature will become apparent below.

To test whether these different conformational states are a result of the applied electric field or some other factor, we collected a smaller cryo-EM dataset on Eag in unpolarized vesicles (i.e., depolarized). Refinement and classification following the same strategy showed that all classes match the up conformation, detailed below (*SI Appendix*, Fig. S4 *A* and *B*). The up map is better defined in the larger polarized vesicle dataset compared with the smaller unpolarized vesicle dataset, so we focus on the former.

### The Up Conformation.

**Fig. 2.**
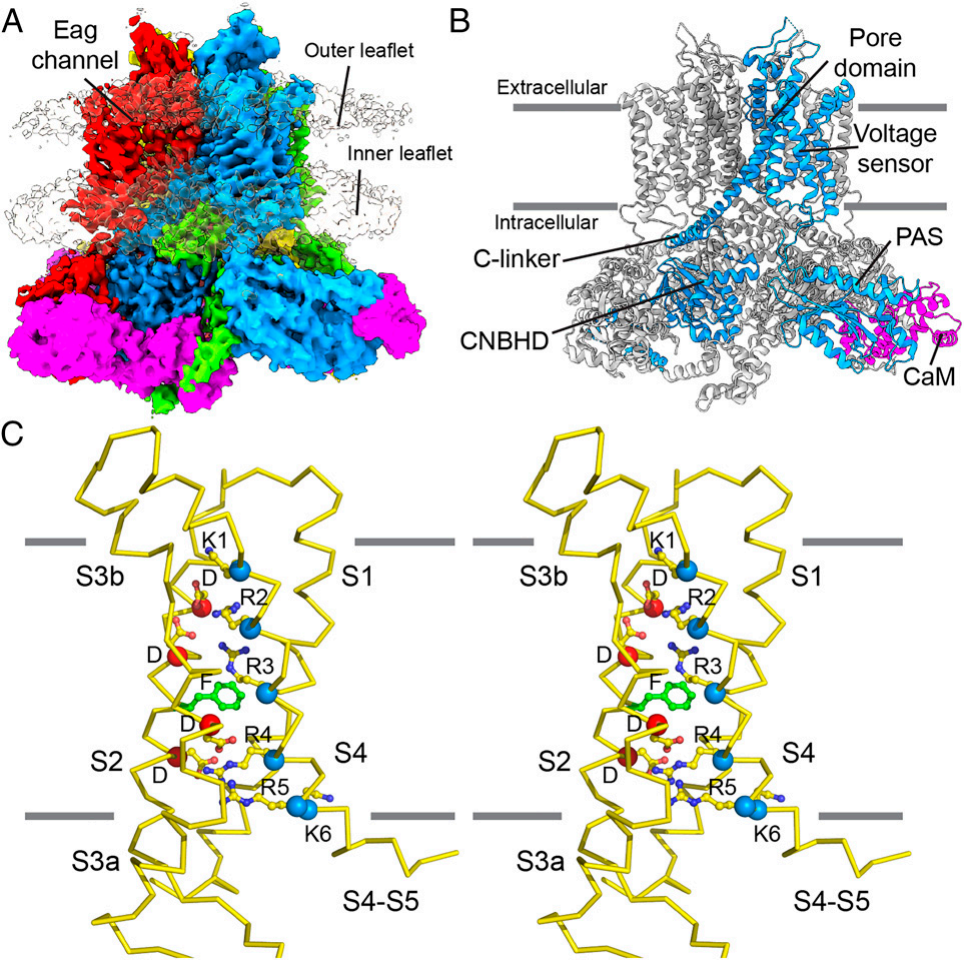
Structure of Eag in a lipid bilayer with the VSD in the up conformation. (*A*) Cryo-EM density map of the Eag channel with each channel subunit shown in a different color and with calmodulin shown in magenta. The two membrane leaflets are shown in the reconstruction at a lower contour. (*B*) Structure of the Eag1 channel (cartoon representation) showing the different domains within one monomer (blue) from the N to C terminus: per-arnt-sim domain, voltage sensor, pore domain, C-linker, cyclic nucleotide-binding homology domain, and the bound calmodulin (magenta). The other monomers are colored gray for clarity. (*C*) Stereo view of the Eag voltage sensor (Cα trace) in the up (depolarized) conformation. The six positive charges in S4 (α-carbon marked by blue spheres), four negative charges in S2 and S3 (D254 and D258 above F261, D264 and D299 below F261, with their α-carbons marked by red spheres), and the hydrophobic phenylalanine in S2 (F261, green sticks) are shown in stick-and-ball representation. CNBHD, cyclic nucleotide-binding homology domain. PAS, per-arnt-sim domain. CaM, calmodulin.

**Fig. 3.**
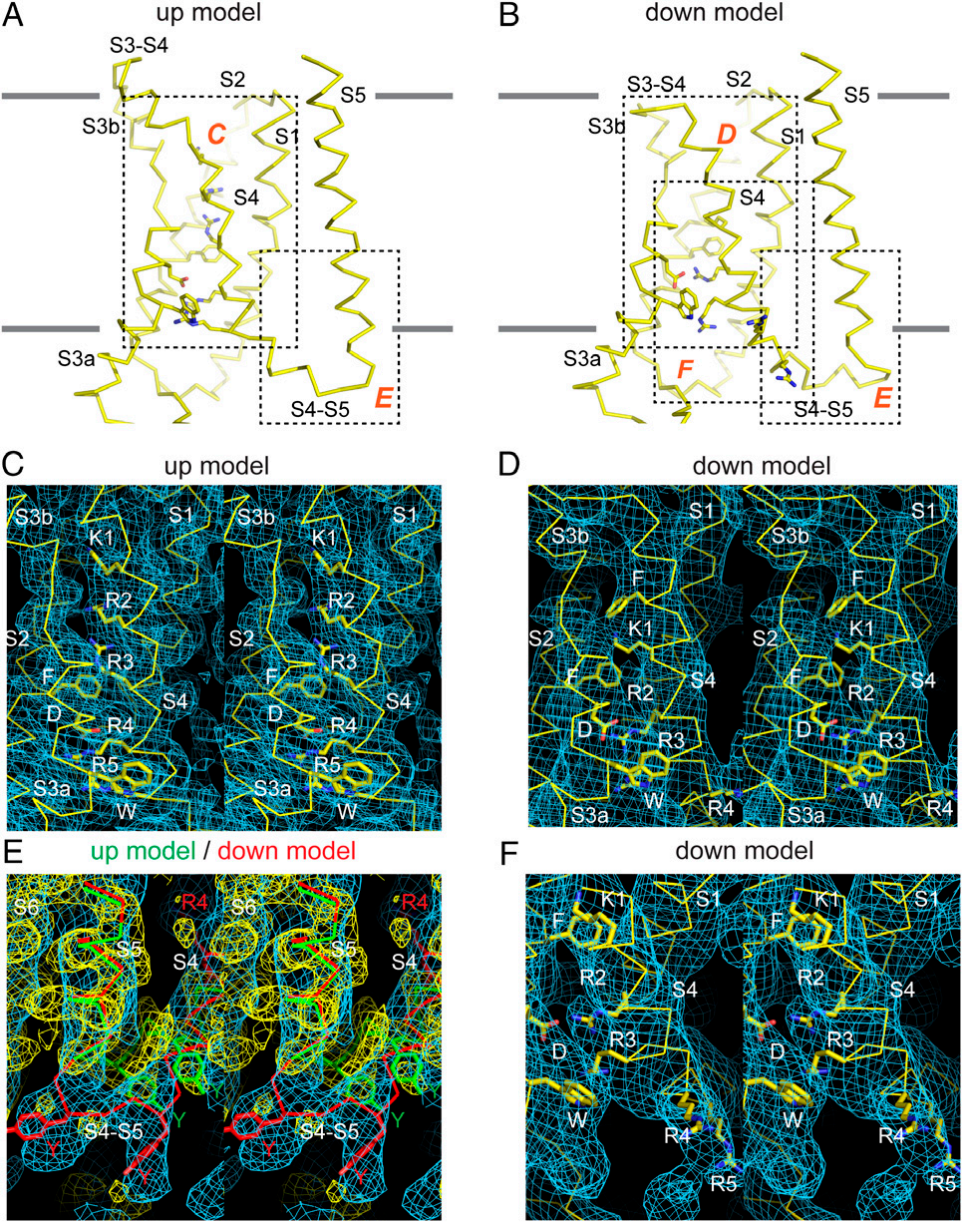
Characterization of electric field–induced movements in the Eag voltage sensor. (*A* and *B*) Cα trace of S1 to S5 in the up model (*A*) and the down model (*B*) highlighting the regions of the cryo-EM density maps shown in the following panels. (*C*) Stereo view of S4 in the up model and map, showing the arginine density above the gating-charge transfer center. The first five positive charged residues and F261, D299, and W295 are shown as sticks for reference. (*D*) Stereo view of S4 in the down model and map showing the location of density for the top of the S4 helix and part of the S3-S4 linker. The first four positive charged residues and F323 (in S4), F261 (in S2), D299, and W295 are shown as sticks for reference. (*E*) Stereo view of the S4-S5 linker region in the up model (green Cα trace) and map (yellow) and in the down model (red Cα trace) and map (blue). Y344, Y347, and R4 (R336) are shown as sticks in both models. (*F*) Stereo view of S4 near the intracellular surface in the down model and map, showing the density attributed to arginine sidechains. R2, R3, R4, and R5 are modeled in the density and K1, F261, D299, and W295 are shown as sticks for reference.

The up map has well-defined density in the TM and enabled building of an essentially complete model including side chains in the voltage-sensor domain (VSD) (Fig. 2 *A*–*C*). The model was built by initially fitting the detergent structure (Protein Data Bank identifier [PDB ID] 5K7L) of Eag with bound calmodulin (18) into the cryo-EM density and making small adjustments where needed. The up structure of Eag in lipid bilayers is very similar to the depolarized structure in detergent micelles, with a slight rotation and translation of the CTD relative to the TM (*SI Appendix*, Fig. S5). The VSD retains the configuration observed in detergent micelles. The S4 is a 3_10_ helix from V328 to L334 and an α helix on either side of this segment. Of the six positive charged residues in the S4 helix, K1 (K327), R2 (R330), and R3 (R333) are positioned at the level of the extracellular membrane leaflet (Fig. 2*C*). Their side chains appear as a continuous strip of density and are positioned to interact with D258 and D254 from S2 (Figs. 2*C* and 3 *A* and *C*). The fourth positive charge, R4 (R336), occupies the gating-charge transfer center inside the membrane. This region of the voltage sensor is formed by F261 from S2, which caps the top of the gating-charge transfer center, and by D264 from S2 and D299 from S3, which interact with the side chain of R4 (Figs. 2*C* and 3*C*). Farther toward the cytoplasm, R5 (R339) is proximal to W295 and D299 in S3, and K6 (K340) is near D221 and W222 in S1 (*SI Appendix*, Fig. S6 *A* and *B*). The pore of the up conformation is tightly closed, as expected of Eag in the presence of Ca^2+^ and calmodulin.

### The Intermediate and Down Conformations.

**Fig. 4.**
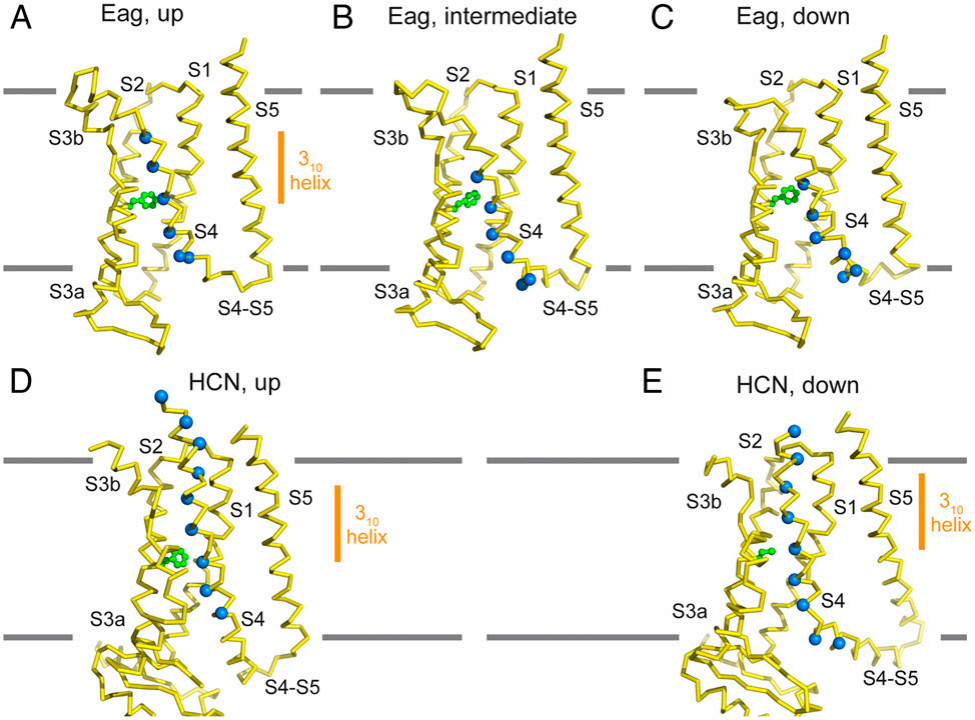
Voltage-sensor conformational changes in Eag and HCN channels. (*A*–*C*) Side view of the Eag voltage sensor in the up (*A*), intermediate (*B*), and the down (*C*) conformations. The Cα positions of the six positive charged residues in S4 are shown as blue spheres, and the gating-charge transfer center residue F261 is shown in green stick-and-ball representation. (*D* and *E*) Side view of the HCN voltage sensor in the up (*D*; PDB ID 5U6P) and the down (*E*; PDB ID 6UQF) conformations (10, 30). The down conformation was stabilized by a metal bridge crosslink. The Cα positions of basic residues and the phenylalanine in the gating-charge transfer center (mutated to cysteine in the down conformation) are shown. All structures are shown in Cα trace representation. The locations of 3_10_ helices in the two HCN structures and the Eag up conformation are marked by orange bars in panels A, D, and E.

The intermediate and down maps are less well defined, no doubt due to conformational heterogeneity. Despite the lower resolution, clear differences in the down map compared with the up map are apparent and were used as constraints to build partial models (Fig. 3 and *SI Appendix*, Fig. S7 *A*–*C*). These differences include an ∼120° rotated S4 to S5 connection and a bent S4, causing S4 to form a more extended interfacial helix near the inner membrane leaflet that, when compared with the up structure, is altered in its length and position relative to S5 (Fig. 3*E*). Concomitant with the appearance of new helical density for S4 near the inner leaflet, there is an approximately equal disappearance of S4 helical density at the membrane’s extracellular surface (Fig. 3 *C* and *D*). Density for many larger side chains also specifies the main chain register. For example, in the down map, density for three arginines is visible: one in the gating-charge transfer center, one below, and one facing the lipid bilayer (Fig. 3*F*), like what is observed above F261 in the up map (Fig. 3*C*). And appearance of the side-chain density below the gating-charge transfer center is concomitant with loss of arginine side-chain density above (Fig. 3 *C*, *D*, and *F*). These and other features of the map constrain K1 to be above the gating-charge transfer center, R2 to be in the gating-charge transfer center, and R3, R4, and R5 to be below the center, rotated toward the periphery of the VSD near the inner leaflet lipid-headgroup layer (Fig. 3*B*). The down structure thus accounts for a net movement of two arginine residues across the gating-charge transfer center, or a two helical turn (∼10 Å) displacement (Fig. 4 *A* and *C*). In addition to the S4 movement, the top half of S3 (S3b) that is directly connected to S4 is also displaced, while S1 and S2 remain in similar positions. All four VSDs in the asymmetric down map show similar densities, which aids map interpretation and model building. Nevertheless, we emphasize that the partial models are built to specify the position and orientation of most residues in S4 rather than to define specific rotamers for residues or exact locations for the main chain atoms.

In the intermediate map, the position of S4 is in between that in the up and down maps, the bend in S4 near the cytoplasmic surface is still apparent, and arginine density is visible below, inside, and above the gating-charge transfer center (*SI Appendix*, Fig. S7*B*). These features constrain K1 and R2 to lie above F261, R3 to be in the gating-charge transfer center, and R4 and R5 to be positioned below the gating-charge transfer center, thus corresponding to the net movement of one arginine or one helical turn (∼5 Å) (Fig. 4*B*). As expected, the pore is closed in the intermediate and down conformations.

### Analogy to the HCN Channel.

In a previous study, stabilization of a down conformation by a metal bridge in the HCN channel showed translocation of two S4 arginine residues across the gating-charge transfer center and formation of an interfacial helix by S4 at the inner membrane leaflet (10), as in Eag (Fig. 4 *D* and *E*). The latter result was unexpected, as the S4 helix was thought to insert into the aqueous solution inside the cell. Instead, toward the bottom of S4, arginine side chains that interact with negative charged residues in the VSD in the up conformation of HCN rotate outward in the down conformation, where they are in position to interact with phospholipid headgroups of the inner membrane leaflet, as in Eag. The formation of an interfacial helix apparently reduces the energetic cost of inserting hydrophobic residues in S4 into the cytoplasm. With displacement of the S4 helix in HCN, residues in the α-helical segment near the extracellular surface reorganize into a 3_10_ helix, while those beginning as a 3_10_ helix reorganize into an α-helix near the intracellular side (Fig. 4 *D* and *E*). The down structure of Eag precludes definitive assignment of α versus 3_10_ helical secondary structure in S4; however, features of the map support a likeness to HCN. Specifically, the outward rotation of the arginine side chains toward the bottom of S4 relative to the side-chain orientation in the gating-charge transfer center is explicable if a 3_10_ helix converts back to an α-helix. Thus, displacement of the Eag S4 likely occurs with an α to 3_10_ “concertina effect” (8), as in HCN (10).

## Discussion

The Eag structures provide a description of voltage-sensor conformational changes that are driven by an electric field generated by a K^+^ gradient across the membrane, like in cells. A voltage sensor in an electric field experiences a fundamentally different set of forces than when it is locked in a specific conformation by a chemical cross-link. In an electric field, a force is exerted on all charged atoms of the system, which includes the protein with its voltage sensors, lipids, and freely diffusing ions. When one thinks about this, it becomes immediately clear that an electric field and a cross-link should not reshape the system identically.

We discuss our findings in the following four sections. 1) The first section considers how movements of the voltage sensors regulate the pore. 2) The second considers the possible extent of voltage-sensor movement in different channels. 3) The third addresses arginine stabilization now that we get to see where the arginines are located in the depolarized and hyperpolarized conformations in the setting of a lipid membrane. 4) In the fourth section, we turn our focus to the membrane near the voltage sensors, to highlight its unexpected change associated with hyperpolarization.

### 1a. A Proposal for Voltage-Dependent Gating in Eag.

**Fig. 5.**
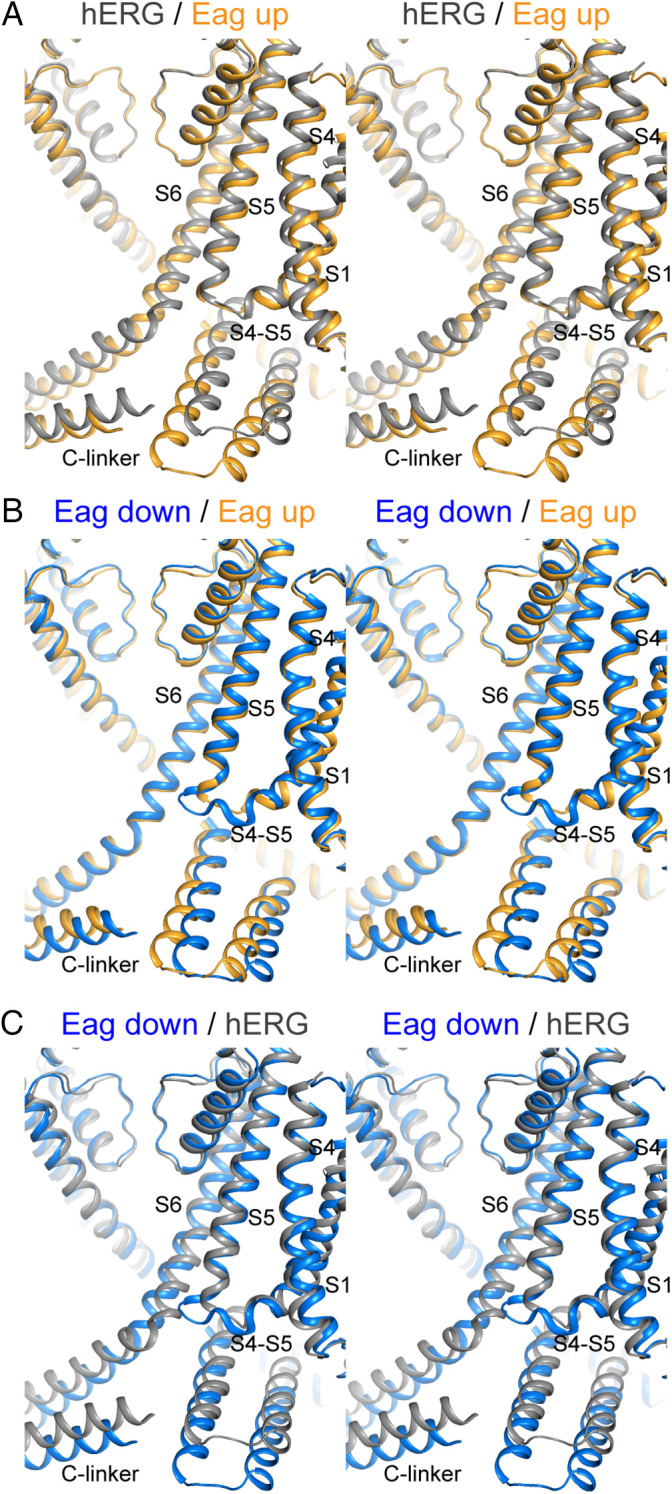
Coupling of voltage-sensor movements in Eag/hERG to pore gating. (*A*) Stereo view of hERG with pore open and S4 up (gray) (17) and Eag with pore closed and S4 up (orange). (*B*) Stereo view of Eag with pore closed and S4 down (blue) and Eag with pore closed and S4 up (orange). (*C*) Stereo view of Eag with pore closed and S4 down (blue) and hERG with pore open and S4 up (gray). The S4 in the down position clashes with the S6 of the same subunit and the C-linker of the adjacent subunit when the pore is open. The channels were aligned using the selectivity filter and the pore helix. The TM domain and C-linker of the subunit with structural elements labeled, the pore domain of the diagonally opposite subunit, and the C-linker of the adjacent subunit are shown in cartoon representation.

We deliberately chose to study voltage-sensor movements in the Eag channel in the presence of Ca^2+^ and calmodulin so that the pore will be held closed when the voltage sensors move. But we have a structure of the hERG K^+^ channel with up voltage sensors and an open pore, and hERG and Eag are very closely related in sequence and structure (17). As shown, their voltage sensors superimpose with high coincidence in the up conformation (*SI Appendix*, Fig. S6*C*), with S4 residues K1, R2, and R3 located above the gating-charge transfer center, R4 inside the gating-charge transfer center, and R5 and K6 below it (Fig. 2*C*). When hERG and Eag are superimposed by aligning their selectivity filters, one can deduce how the pore of Eag can open by adopting the conformation observed in hERG (Fig. 5*A* and Movies S1 and S2). The pore lining S6 helices expand outward (away from the pore axis) by an ∼5-Å radius. The expansion is associated with a similar outward displacement of the S5 helix and a rotation of the C-linker. When these changes are enacted in the up conformation of Eag, no steric clashes occur among S6, the C-linker, and the pore. Therefore, we posit that if calmodulin were not holding the pore of Eag closed through rotation of the C-linkers, then the pore would spontaneously adopt the open conformation observed in the hERG channel.

In the down conformation of Eag, the S4-S5 linker, now a longer interfacial helix, slides beyond S5 in the triangular cleft between S5, S1, and the C-linker helices (Fig. 5*B* and Movies S1 and S2). The functional consequence of this new position of the voltage sensor becomes apparent when the down conformation of Eag is superimposed on the open hERG channel (Fig. 5*C* and Movies S1 and S2). Steric clashes occur between the hERG’s open S6 helix and the down S4-S5 linker of Eag from the same subunit and C-linker from an adjacent subunit. The S6 cannot push the S4-S5 linker out of the way because the latter is buttressed against S1 (Fig. 5*C* and Movies S1 and S2). Thus, the down position of S4 precludes pore opening by clamping down on the S6 helix bundle gate. This explains why the pore of Eag can open at depolarized membrane voltages but not at hyperpolarized voltages, in which case four S4-S5 linkers, made longer by the down conformation of the voltage sensors, form a constrictive cuff around the closed S6 helix gate.

To imagine the sequential event path of voltage-sensor regulation of the pore, we have made movies by interpolating between the closed Eag with down voltage sensors, the closed Eag with up voltage sensors, and the open hERG with up voltage sensors (Movies S1 and S2). Beginning in a hyperpolarized membrane (i.e., at the resting membrane voltage of a cell), depolarization drives the voltage sensors up, which draws the S4-S5 linkers away from the closed S6 helices, permitting them to open spontaneously. The sequence of events shown are consistent qualitatively with electrophysiological studies on several voltage-dependent ion channels, which show that upon depolarization from a negative membrane voltage, transient gating currents associated with the movement of charges in the voltage sensors occur first, followed by ionic currents when the pore subsequently opens (9, 12, 31).

### 1b. The Possible Origin of a Voltage-Dependent Polarity Switch.

We described similarities in the voltage-sensor conformational changes observed in HCN (mediated by a metal bridge) and in Eag (mediated by voltage) (Fig. 4), but these two channels exhibit opposite gating polarities: Eag is opened by membrane depolarization, while HCN is opened by membrane hyperpolarization (32). How can similar conformational changes in the voltage sensors have opposite effects on the pore? In Eag/hERG, which are depolarization-activated channels, the down conformation of the voltage sensor prevents pore opening by increasing the contact between the S4-S5 linker and S1, the closed pore, and the C-linker (Fig. 5 and *SI Appendix*, Fig. S8*A*). By contrast, the down conformation of HCN’s voltage sensor decreases the contact. The main reason for this is in Eag: as the S4 helix moves down, it slides past S5 and closer to S1 and the C-linker. In HCN, S4 is ensconced by S5, S1, and the C-linker in the up conformation. As the S4 helix moves down, instead of moving past S5, it pushes S5 to disengage S6, which causes S4 to move further from S1 and the C-linker (*SI Appendix*, Fig. S8*B*). Thus, it appears that contacts between the voltage sensor and the pore are diminished in the down conformation of HCN. These observations seem to imply that voltage sensors in depolarization-activated channels relieve their inhibition on the pore in the up conformation, while the opposite appears true in hyperpolarization-activated channels. We note that in the HCN channel locked in a down conformation by a cross-link, the pore did not open (10). We suspect that, in HCN, the voltage sensor may have to move farther down to permit pore opening.

### 1c. Domain-Swapped versus Nondomain-Swapped Voltage Sensors.

Eag and hERG, as well as HCN and Slo1, are called “nondomain-swapped” voltage-dependent ion channels (17, 18, 30, 33). This term means that the VSD is located adjacent to a pore subunit formed by the same polypeptide chain. By contrast, “domain-swapped” voltage-dependent ion channels, which include K_v_1 through 7 and voltage-dependent Na^+^ and Ca^2+^ channels (8, 11, 14, 15, 19, 34–36), have VSDs adjacent to a neighboring pore subunit. The overall architecture of these two classes of voltage-dependent ion channels is very similar, especially the VSDs; however, the domain-swapped arrangement in the latter class requires a long interfacial helix called the S4-S5 linker helix. Because domain-swapped voltage-dependent ion channels have permanent interfacial helices, so-called S4-S5 linker helices, surrounding the pore even when the VSD is up, our data on Eag do not directly address how VSDs regulate those channels.

### 2. Gating-Charge Magnitudes.

**Fig. 6.**
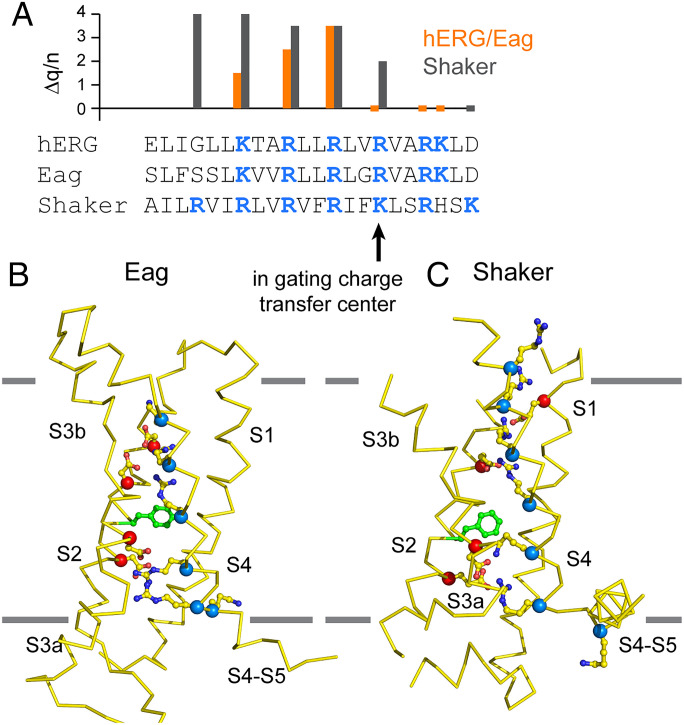
Implied voltage-sensor movements in Shaker compared with Eag. (*A*) Sequence alignment of the S4 segment of the Eag, hERG, and Shaker K^+^ channels based on their structures (36). The positive charged residues are colored blue. Shown above the sequences is a plot of the change in the gating-charge per channel (Δq/n) associated with charge-neutralizing point mutations in hERG (37), where Δq/n was estimated using the limiting slope method (orange), and in Shaker, where Δq/n was estimated by nonlinear membrane capacitance (black) (38). The residue that occupies the gating-charge transfer center in the up conformations in each channel is marked by a black arrow in the sequence. (*B* and *C*) Side view of the Eag (*B*) and Shaker (*C*; PDB ID 7SIP) voltage sensors (Cα trace) in the up (depolarized) conformation. The Cα positions of positive and negative charges are shown as blue and red spheres, respectively, and side chains for these residues are shown in stick-and-ball representation. The hydrophobic phenylalanine is shown in green stick-and-ball representation.

All voltage-dependent ion channels contain the (RXX)_n_ triplet in their S4 helices’ however, n is variable, as is the magnitude of gating charge calculated from gating-current measurements. It is useful to compare Eag and hERG with the well characterized Shaker K^+^ channel. A sequence alignment for the S4 helix of these channels is shown in Fig. 6*A* and the voltage sensor structures in the up (depolarized) conformation are shown in Fig. 6 *B* and *C* (36) (See also *SI Appendix*, Fig. S6*C*). In the depolarized conformation, Eag and hERG both have three positive charges (K1, R2, and R3) above, one (R4) inside, and two (R5 and K6) below the gating-charge transfer center (*SI Appendix*, Fig. S6*C*). Two clusters of negative charged residues, one formed by two aspartates near the extracellular side and another in the gating-charge transfer center, are likely occupied by a positive charged residue to maintain a degree of charge balance, except during brief transitions between conformations driven by voltage (Fig. 6*B*). Charge balance would tend to hold K1 above F261 to interact with D258 and D254 during hyperpolarization, as we observe in the down structure of Eag, limiting the displacement to two charges crossing the gating-charge transfer center (Fig. 4 *A*–*C*). We note that gating-charge estimates from limiting slope analysis in hERG, assumed to be like Eag, imply that six elementary charges per channel (or 1.5 per voltage sensor times 4) cross the membrane voltage difference during channel activation (37). Keeping in mind that the electric field is unlikely to be constant across the voltage sensor, the gating-charge estimate from limiting slope measurements in hERG and the physical displacement of charges in the structures of Eag are in rather good agreement.

The Shaker channel, on the other hand, has an additional positive charge in its S4, with four above, one in, and two below the gating-charge transfer center in its up conformation (Fig. 6*C*) (36). Following the reasoning above, we might expect in the down conformation that R1 will reside near the negative charges above F261 in Shaker. This would yield a displacement of three charges per sensor, in line with gating-charge estimates of 12 to 14 per channel (or 3 to 3.5 per voltage sensor) (38–40). Thus, we might expect the Shaker channel S4 to move three helical turns, or ∼15 Å.

While the above description is certainly oversimplistic, functional measurements are supportive. In both Shaker and hERG, the estimated gating charge per channel (∼13 and ∼6 charges, respectively) is less than the sum of reduced charges (Fig. 6*A*) when each positive charged residue is mutated to a neutral residue (∼18 and ∼7.5 charges, respectively) (37, 38). If a mutation affected only the charge contributed by the mutated site and no other aspect of the protein, the sum of reduced charges would be less than or equal to the total gating charge. Moreover, neutralization of K1 in hERG reduces total gating charge by 1.5, yet neither our structure nor the total gating-charge estimates support K1 itself translocating across the gating-charge transfer center. To make this point clearer, when the charge on K1 is removed by mutation, charge balance will likely bias R2 to remain above F261; consequently, it will contribute less to the total gating charge. Similar reasoning can explain why a mutation of R1 in Shaker alters the gating charge to such an extent and why mutation of Asp and Glu residues surrounding S4 can alter the gating charge (37, 40). Thus, it becomes clear that mutations removing a positive-charge affect total measured gating charge for two reasons: first, the number of charges on S4 is reduced, and second, the total displacement of S4 changes. With these ideas in mind, the change in total gating charge resulting from S4 mutations (Fig. 6*A*) suggests that the S4 in Shaker displaces to a greater extent than in Eag or hERG.

### 3a. The Stabilization of Arginine Side Chains.

In all three maps (i.e., up, intermediate, and down), continuous, sheet-like density for arginine (and lysine) residues is observed, but the location of this density varies. In the up conformation, two arginines and one lysine form such an assembly above F261 (Fig. 3*C* and *SI Appendix*, Fig. S7*A*), while the intermediate (*SI Appendix*, Fig. S7*B*) and down (Fig. 3*F* and *SI Appendix*, Fig. S7*C*) maps show density with a similar appearance below F261 that we attribute to arginines. What interactions might stabilize these assemblies of basic residues? In addition to the negative charged aspartates and glutamates that form salt bridges, we hypothesize that water molecules can intercalate in between and stabilize the guanidinium groups by facilitating delocalization of the positive charge through hydrogen bonds. While the resolution of our cryo-EM maps blur these details, such water molecules are indeed visible in the 2.4-Å crystal structure of K_v_1.2 paddle chimera (8). The conservation of these sheets of density in the intermediate and down maps suggests that similar chemistry could stabilize these arginine assemblies in both depolarized and hyperpolarized conformations.

### 3b. The Influence of Phospholipids on Channel Gating.

Phospholipids play an important role in the function of certain voltage-gated potassium channels. For example, the archeal K_v_AP channel requires phospholipids in the membrane to open; it remains completely inactive in nonphospholipid membranes (41). The lipid phosphatidic acid (PA) was found to directly influence channel gating, both in a domain-swapped channel (K_v_1.2 paddle chimera) and a nondomain-swapped channel (K_v_AP) (42). Adding PA in planar bilayers shifted the voltage activation midpoints of these channels to more depolarizing potentials by ∼20 to 30 mV. The PA effect is specific to the inner leaflet of the membrane and involves arginine residues in S4, suggesting that ionized hydrogen bonding between arginine guanidinium and primary phosphate groups could stabilize the closed state of channels (or voltage-sensor down conformations). The voltage sensor down conformations observed here and previously in HCN show that arginine residues translocate downward and rotate outward, such that their guanidinium side chains are poised to interact with phosphate headgroups in the inner leaflet of the membrane. Favorable interactions between R4 and R5 and phosphate headgroups could thus stabilize the closed state in Eag. We note that this prediction is specific to residues near the bottom of S4 (i.e., not all arginine residues would be exposed to the headgroup region for a given voltage sensor). Indeed, in K_v_AP, the PA-specific channel gating effect is observed only for the two positive charged residues nearest the intracellular side, while mutation of residues further toward the extracellular side preserves the PA effect, lending further credence to this notion (42). In summary, the influence of anionic phospholipids on K_v_ channel gating can be explained by the favorable interactions with S4 arginine residues when they become membrane exposed in the down conformation.

### 4. Membrane Thinning in the Down Conformation.

**Fig. 7.**
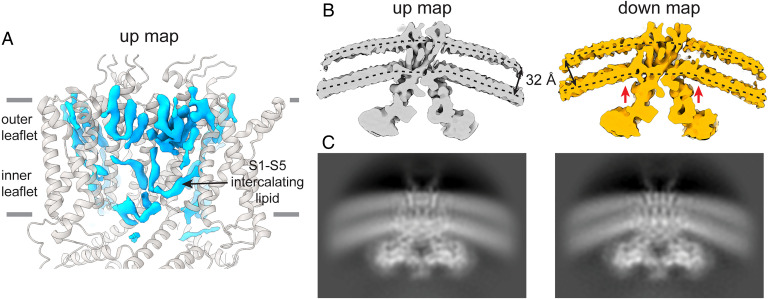
Voltage-sensor effects on the lipid membrane. (*A*) Lipid densities in the outer and inner leaflets visible in the up map. The densities are better defined in the outer leaflet compared with the inner leaflet. (*B* and *C*) Cross-sections (*B*) and two-dimensional projections (*C*) of 7-Å low-pass–filtered up (*Left*) and down (*Right*) maps. Dotted lines represent the approximate midpoints of the outer and inner leaflet headgroup densities in the up map, which are separated by ∼32 Å. These reference lines are replicated on the down map drawn to the same scale and show that the membrane is thinned near the channel (red arrows), owing to a ∼5 Å displacement of the inner-leaflet headgroup layer.

By solving the structures of Eag in lipid-bilayer vesicles, we can directly look at the membrane surrounding a K_v_ channel in different conformational states. The up map shows nonprotein densities consistent with associated lipid or sterol molecules that are better defined in the outer leaflet compared with the inner leaflet (Fig. 7*A*). The lipids in the inner leaflet can adopt poses that are not normal to the bilayer, such as an acyl chain angled between S1 and S5. These observations are also apparent in the crystal structure of K_v_1.2 paddle chimera (8), suggesting that the inner leaflet is generally more perturbed in the vicinity of a K_v_ channel.

More striking is an apparent thinning of the membrane surrounding the channel in the down map. Cross-sections (Fig. 7*B*) and 2D projections (Fig. 7*C*) of low-pass–filtered up and down maps show strong density for phosphate headgroups in the outer and inner leaflets that are separated by ∼32 Å. But in the down map, near the channel, the inner leaflet density moves closer to the outer leaflet by ∼5 Å. Toward the edges of both maps, the bilayer thickness is equal, but the headgroup density becomes broader, reflecting the distribution of vesicle sizes in our preparation.

What might cause this membrane thinning in the down conformation? It is possible that the hydrophobic surface of the protein is reshaped as S4 moves down, which might distort the membrane through hydrophobic matching. The arginines that are oriented toward the membrane in the down conformation might also hold phospholipids in particular orientations, or attract certain types of lipids (i.e., cause lateral segregation). Another explanation is that the applied voltage causes constriction of the charged phosphate-head-group layer very close to the channel. While we do not yet know the reason for the observed thinning, the implications for voltage sensing are apparent: it will focus the electric field, in a conformation-dependent manner, to increase the electrostatic force experienced by basic residues in S4. It has been proposed before that the electric field is focused in both hyperpolarized and depolarized conformations of the voltage sensor through water-filled cavities (43, 44). The conformation/voltage-dependent thinning of the membrane around the channel is a different phenomenon. Further studies are needed to understand the mechanism of this membrane thinning and the consequences for voltage sensing.

### Distribution of Voltage Sensor Conformations.

The reader may wonder why half the Eag channels still have their voltage sensors in the up conformation when the voltage difference across the membrane is so negative, apparently producing a mean electric field of ∼3 × 10^7^ V/m. There are at least two reasons we can think of that may account for this. First, we emphasize that the estimation of −145 mV represents an upper limit: the actual membrane potential might be lower due to leak of Na^+^ or Cl^−^. It is also possible that some vesicles are more permeable to these ions than others, leading to a distribution of potentials. Second, cell membranes have different lipid compositions in their inner and outer leaflets, while our synthetic liposomes do not. In the plasma membrane, the anionic lipid phosphatidylserine comprises ∼20% of the total phospholipids and normally resides only in the inner leaflet (45). In other words, the inner leaflet is composed of 40% negative charged phosphatidylserine while the outer leaflet contains practically no charged lipids. This lipid asymmetry is thus expected to contribute significantly to the electric field inside the membrane via a surface-charge voltage offset that augments the directly applied membrane potential (e.g., in electrophysiological measurements) (42). The magnitude of this offset is approximately −75 mV for 150 mM of a monovalent electrolyte such as KCl and assuming a lipid cross-sectional area of 0.65 nm^2^ (46). This surface potential is substantial in the realm of voltages we are concerned with. Finally, we note that the surface-charge effect described above is independent of the chemical interaction effect described for PA in the previous section, and thus can be additive. It should still be possible to drive the conformational equilibrium of the voltage sensors so that nearly all of them are in a uniform down conformation, but more sophisticated experiments might be required.

## Materials and Methods

### Cell Lines.

Sf9 cells were used for production of baculovirus and were cultured in Sf-900 II SFM medium (GIBCO) supplemented with 100 U/mL penicillin and 100 U/mL streptomycin at 27 °C.

HEK293S GnTl^−^ cells were used for protein expression and were cultured in Freestyle 293 medium (GIBCO) supplemented with 2% fetal bovine serum, 100 U/mL penicillin, and 100 U/mL streptomycin at 37 °C in 8% CO_2_.

### Expression and Purification of Eag1 Bound to Calmodulin.

The Eag1-calmodulin complex was expressed and purified as described before (18), with slight modifications. We used a construct corresponding to rat Eag1 with residues 773 to 886 truncated (rEag1), cloned into the BacMan expression vector with a C-terminal green fluorescent protein (GFP)–His_6_ tag linked by a preScission protease site (47). A separate BacMan expression vector without a tag was used for vertebrate calmodulin (CaM).

Bacmids were generated for rEag1 and CaM with DH10Bac *Escherichia coli* cells. Baculoviruses for rEag1 and CaM were produced in SF9 cells transfected with bacmid DNA using the Cellfectin II reagent (Invitrogen). Baculovirus was amplified three times in 1-L suspension cultures of SF9 cells at 27 °C. We infected 4-L suspension cultures of HEK293S GnTI^−^ at ∼3 × 10^6^ cells/mL with 12% (vol/vol) of 5:1 rEag1:CaM baculovirus at 37 °C for 10 h. Protein expression was induced by adding 10 µM sodium butyrate, and the growth temperature was changed to 30 °C. Cell pellets were harvested 48 h postinduction and flash frozen in liquid nitrogen for later use.

We resuspended 4 L of cell pellet in ∼50 mL of lysis buffer (25 mM Tris at pH 8.0, 300 mM KCl, 1 mM MgCl_2_, 5 mM CaCl_2_, 2.5 mM dithiothreitol [DTT], 1 µg/mL leupepetin, 1 µg/mL pepstatin, 1 mM benzamidine, 1 µg/mL aprotonin, 1 mM phenylmethylsulfonyl fluoride, 1 mM 4-(2-aminoethyl)benzenesulfonyl fluoride, and 0.1 mg/mL DNase), stirred for 10 min at 4 °C, and dounce homogenized with a loose pestle until homogenous. The resultant suspension was clarified by centrifugation at 39,800*g* for 15 min at 4 °C. The pellet was resuspended in ∼100 mL of lysis buffer and dounce homogenized with a tight pestle. To extract the complex, we added 15 mL of a 10%:2%:0.13% *n*-dodecyl-β-d-maltopyranoside (DDM) to cholesteryl hemisuccinate (CHS) to lipids mixture [wt/vol in distilled H_2_O] and stirred for 1 h at 4 °C. The lipids used were 3:1:1 POPC to 1-palmitoyl-2-oleoyl-*sn*-glycero-3-phosphoethanolamine (POPE) to POPG [wt/wt/wt].

The mixture was clarified by centrifugation at 39,800*g* for 30 min at 4 °C, and the supernatant was bound to ∼2.5 mL of GFP nanobody-coupled Sepharose resin (prepared in house) (48) for 1 h. The resin was washed with ∼40 column volumes of wash buffer (20 mM Tris at pH 8.0, 300 mM KCl, 0.1%:0.02% DDM to CHS, 0.1 mg/mL lipids, 1 mM CaCl_2_, and 2.5 mM DTT). The resin was resuspended in five-column volumes of wash buffer, preScission protease (prepared in house) was added at a concentration of 0.05 mg/mL to remove the GFP tag, and the solution was rotated gently for 1.5 h at 4 °C. The cleaved protein was collected in the flow through and a subsequent wash step with a five-column volume of wash buffer. The protein was concentrated to ∼700 µL at 3,500*g* and 4 °C using a 15-mL Amicon spin concentrator with a 100-kDa molecular weight cutoff membrane. The concentrated protein was filtered through a Corning 0.2-µm spin filter and then purified by size-exclusion chromatography using a Superose 6 Increase column (10/300 GL) preequilibrated with size-exclusion chromatography buffer (10 mM Tris at pH 8.0, 300 mM KCl, 0.05%:0.01% DDM to CHS, 0.05 mg/mL lipids, 0.1 mM CaCl_2_, and 5 mM DTT). Fractions containing Eag and calmodulin were pooled and concentrated to an A_280_ of 3 mg/mL at 3,000*g* and 4 °C using a 4-mL Amicon spin concentrator with a 100-kDa molecular weight cutoff membrane. Purified protein was immediately used for reconstitution into liposomes.

### Reconstitution of the Eag1-CaM Complex into Liposomes.

Lipids (90%:5%:5% POPC to POPG to cholesterol [wt/wt/wt], all from Avanti) were mixed together in chloroform at a concentration of 10 mg/mL and dried to a thin film under a gentle stream of argon. The lipid film was further dried for 4 h in a room-temperature vacuum desiccator and then resuspended at a concentration of 10 mg/mL by gentle vortexing in reconstitution buffer (10 mM Tris at pH 8.0, 300 mM KCl, and 2 mM DTT). Small unilamellar vesicles were formed by bath sonication at room temperature until the solution was mostly transparent, typically ∼20 min. To permeabilize but not solubilize the vesicles, the detergent C_12_E_10_ was added to the lipid stock solution to a final concentration of 2 mg/mL and incubated on ice for 30 min. We mixed 200 µL of this permeabilized vesicle solution with 25 µL of purified Eag1-CaM (3 mg/mL) and 175 µL of reconstitution buffer, giving a total reaction volume of 400 µL, a protein to lipid ratio of 1:27 (wt/wt), and a final lipid concentration of 5 mg/mL. A low protein to lipid ratio was used to minimize protein aggregation and detergent in the sample. The lipid-protein-detergent mixture was incubated on ice for 1.5 h. Detergent was removed using adsorbent Bio-Beads SM-2 resin (Bio-Rad) by adding 20 mg of a 50% (wt/vol) Bio-Beads slurry in reconstitution buffer and rotating at 4 °C for 12 h. The removal procedure was repeated twice again for 3 h each. Proton NMR spectroscopy was used to confirm quantitative recovery of lipids and sterol and practically complete detergent removal in the preparation. For the unpolarized vesicles, we used 150 mM KCl (instead of 300 mM) and a higher protein to lipid ratio of 1:14 (wt/wt). The single-step reconstitution protocol described here permitted efficient incorporation of Eag-CaM into liposomes for cryo-EM analysis.

### Preparation of Polarized Vesicles and Cryo-EM Grids.

To the proteoliposomes prepared above, 2 µM valinomycin (from an 8-mM stock in dimethyl sulfoxide) was added and incubated for 30 min on ice. We added 70 µL of this solution to a 0.5-mL Zeba spin desalting column (Thermo Scientific) with a 40-kDa molecular weight cutoff, preequilibrated with sodium reconstitution buffer (10 mM Tris at pH 8.0 and 300 mM NaCl). The sample was centrifuged for ∼30 s at room temperature at 1,500*g* and ∼20 µL of flow-through containing vesicles was collected. We immediately applied 3.5 µL of the polarized vesicle solution onto a glow discharged Quantifoil R1.2/1.3 400 mesh holey carbon Au grid. After incubating the sample on the grid for 3 min at 20 °C with a humidity of 100%, the grid was manually blotted using a filter paper. Another 3.5 µL of the polarized vesicle solution was applied to the same grid for 20 s (49), and then the grid was blotted for 3 s with a blotting force of 0 and flash frozen in liquid ethane using an FEI Vitrobot Mark IV. Each grid used a freshly buffer-exchanged sample and the delay between starting the buffer exchange and the sample being frozen was ∼4 min. The grids for the unpolarized vesicles were frozen directly after reconstitution (i.e., without valinomycin or buffer exchange).

### Measurement of Potassium Concentration after Buffer Exchange.

The residual external potassium concentration after buffer exchange was measured externally (Applied Technical Services) by inductively coupled plasma-optical emission spectrometry. In two samples with known K^+^ concentrations of 1 mM and 10 mM, the measured K^+^ concentrations were 1 mM and 6 mM, respectively, validating the method. In two test samples, one of which was an average of four buffer-exchanged proteoliposome samples used for freezing grids, and the second being a buffer-exchanged sample without vesicles, the potassium concentrations were measured to be 1 mM.

### Liposome Flux Assay.

The flux assay was carried out as described before (29), with minor modifications. The proteoliposome vesicles or control vesicles without protein prepared in 300 mM KCl were diluted 10-fold in isotonic sodium buffer (10 mM Tris at pH 8.0, and 300 mM NaCl) immediately prior to the assay. We mixed 6 µL of the diluted vesicle solution with 6 µL of ACMA solution (10 mM Tris at pH 8.0, 300 mM NaCl, and 5 mM ACMA) and 12 µL of buffer (10 mM Tris at pH 8.0, and 300 mM NaCl). ACMA fluorescence was recorded every 5 s (excitation wavelength = 410 nm; emission wavelength = 490 nm) using a 384-well plate (Grainger) on a fluorescence plate reader (Tecan Infinite M1000). After the ACMA fluorescence stabilized, 6 µL of CCCP solution (10 mM Tris at pH 8.0, 300 mM NaCl, and 15 mM CCCP) was added. The resultant Eag-dependent flux, or in this case, the lack thereof, was measured. At the end of the assay, 2 µL of a 1.2 µM valinomycin solution (in 10 mM Tris at pH 8.0, and trace dimethyl sulfoxide) was added to initiate K^+^ efflux from all the vesicles and determine the minimum ACMA fluorescence. The fluorescence data for each run were normalized by the fluorescence value right before addition of CCCP (i.e., at 90 s). The normalized data were averaged across three independent measurements and the mean and SDs are reported.

### Cryo-EM Data Acquisition and Processing.

Grids for the polarized vesicles were loaded onto a 300-keV Titan Krios 2 transmission electron microscope equipped with a Gatan K3 detector. A total of 25,794 movies were recorded on a single Quantifoil grid in super-resolution mode with a 100-µm objective aperture using SerialEM (50). The movies were recorded with a physical pixel size of 1.08 Å (super-resolution pixel size of 0.54 Å) and a target defocus range of −1.0 to −2.0 µm. The total exposure time was 1.6 s (0.04 s/frame) with a dose rate of 40 e^−^/pixel/s, which gave a total cumulative dose of 56 e^−^/Å^2^ (1.4 e^−^/Å^2^/frame).

The data processing workflow is summarized in *SI Appendix*, Fig. S2. Initial data processing was carried out in cryoSPARC version 3.3.1 (51). The super-resolution movies were gain-normalized, binned by a factor of 2 with Fourier cropping, and corrected for full-frame and sample motion using the Patch motion correction tool (grid = 15 × 10). Contrast transfer function (CTF) parameters were estimated from the motion-corrected micrographs using the Patch CTF estimation tool, which uses micrographs without dose weighting. All subsequent processing was performed on motion-corrected micrographs with dose weighting.

Particle picking was initially carried out using the Blob picker and Template picker tools. For the latter, a previously acquired 3D reconstruction of Eag1-CaM in liposomes was used. After two or three rounds of 2D classification, classes with clear protein density were visible. Approximately 5% of these particles and micrographs were also used to train a TOPAZ (52) picking model, which was used to pick additional particles. Particles with clear protein density after 2D classification were pooled and duplicate picks were removed. A total of 653,450 particles were used for subsequent 3D classification in classification round 1A (see *SI Appendix*, Fig. S2).

The selected particles were refined in cryoSPARC using a previously obtained reconstruction of Eag in liposomes. Ab initio reconstruction followed by refinement gave similar results. This initial reconstruction was used along with “junk” particle reconstructions in one round of heterogeneous refinement in cryoSPARC. One class with 322,496 particles had strong protein density and was further locally refined with a mask on the pore and CTD, excluding the VSD. The particles in this reconstruction were subjected to focused classification without alignment in RELION 3.1 (53), using a mask on the TM while excluding the CTD. Only two classes had well-defined pore density as well as visible voltage sensor density. One of these two classes was consistent with the detergent structure of Eag (i.e., the up conformation), while the second one appeared different in the S4-S5 linker region.

The first class with 54,654 particles corresponds to the Eag up conformation. Local refinement was performed in RELION with C4 symmetry imposed. This map was essentially identical to the final up map, with slightly lower nominal resolution and map quality.

The second class with 50,698 particles corresponds to the Eag down and intermediate conformations. After local refinement using C4 symmetry, we performed symmetry expansion of the particles to get 202,792 particles. Two rounds of 3D classification without alignment (C1 symmetry) were used to identify a class with the most distinct VSDs. This class with 31,642 symmetry-expanded particles (labeled “Intermediate/Down particle set 1” in *SI Appendix*, Fig. S2) was selected for round 2 of classification.

To improve the quality of these maps, we repeated the same classification strategy on a slightly bigger particle set (starting with 808,521 after 2D classification; classification round 1B). After two rounds of heterogeneous refinement in cryoSPARC, one class with 258,782 particles was further refined with a mask on the pore and CTD, excluding the VSD. As in the earlier round, we performed classification on the TM without alignment in RELION 3.1. One class with 17,187 particles had a different S4-S5 linker position compared with the up conformation, like that observed in Intermediate/Down particle set 1. We performed local refinement using C4 symmetry, symmetry expansion of the particles, and then 3D classification without alignment (C1 symmetry) to identify the best class with 25,689 particles, termed “Intermediate/Down particle set 2.”

For the second round of classification, we pooled the intermediate/down particles from the two classifications and removed duplicates (including symmetry-related particles), giving a total of 30,506 unique particles. Global refinement was performed using C4 symmetry. Then we performed symmetry expansion followed by 3D classification without alignment (C1 symmetry) to identify one class with 36,217 particles that corresponds to the down map. We performed local refinement without symmetry in cryoSPARC and sharpened the map using a B-factor of −50. The intermediate map was obtained using a different class from the above classification, after an additional round of classification. A total of 33,914 particles were used to calculate this map (using C1 symmetry) in cryoSPARC, and no sharpening was performed.

The final up map was calculated as follows. We pooled all particles that had reasonable pore and voltage-sensor density in the very first classification from both rounds (1A and 1B). Duplicates were removed, and then we conducted global refinement using C4 symmetry. We performed classification without alignment on the TM. A single best class with 52,134 particles was selected for further refinement. We removed particles in this set that were also present in the intermediate/down particle sets 1 and 2, and then performed local refinement using C4 symmetry on the final 49,164 particles in cryoSPARC. Per-particle local CTF refinement was used, followed by local refinement to obtain the final map. Map sharpening was performed using the B-factor of -116 estimated from the Guinier plot.

Grids for the unpolarized vesicles were loaded onto a 300-keV Titan Krios transmission electron microscope equipped with a Gatan K2 Summit detector. A total of 1,674 movies were recorded on a single Quantifoil grid in super-resolution mode with a 100-µm objective aperture using SerialEM. The movies were recorded with a physical pixel size of 1.33 Å (super-resolution pixel size of 0.665 Å) and a target defocus range of −1.0 to −2.0 µm. The total exposure time was 12 s (0.24 s/frame) with a dose rate of 10 e^−^/pixel/s, which gave a total cumulative dose of 69 e^−^/Å^2^ (1.38 e^−^/Å^2^/frame). Data processing was performed as for the polarized vesicles.

### Model Building and Refinement.

An initial model was built by docking the structure of Eag1-CaM in detergent micelles (PDB ID: 5K7L) into the cryo-EM density map for the up conformation. Adjustments were only made where the existing structure did not fit in the cryo-EM density map. The model was edited and refined using the ISOLDE (54) plugin in ChimeraX, version 1.2.0 (55), or WinCoot, version 0.98.1 (56), followed by real-space refinement in Phenix (57). The down and intermediate models were built starting from the up model and following a similar protocol. The quality of the final models was evaluated using the MolProbity plugin in Phenix. Graphical representations of models and cryo-EM density maps were prepared using PyMOL (58) and ChimeraX.

## Supplementary Material

Supplementary File

Supplementary File

Supplementary File

## Data Availability

Cryo-EM density maps of the Eag channel with the voltage sensor in the up, intermediate, and down conformation have been deposited in the Electron Microscopy Data Bank under accession codes EMD-28487 (59), EMD-28494 (60), and EMD-28498 (61), respectively. Atomic coordinates of the Eag channel with the voltage sensor in the up, intermediate, and down conformation have been deposited in the Protein Data Bank under accession codes 8EOW (62), 8EP0 (63), and 8EP1 (64), respectively.
