# Supplementary material for: Voltage-sensor movements in the Eag Kv channel under an applied electric field
Source: Proc Natl Acad Sci U S A. 2022 Nov 7;119(46):e2214151119. doi: 10.1073/pnas.2214151119 (PMC9674223; doi:10.1073/pnas.2214151119)
Supplement: Supplementary File [file pnas.2214151119.sapp.pdf]

PNAS

Supporting Information for

Voltage sensor movements in the Eag Kv channel under an applied electric field.

Venkata Shiva Mandala[1] and Roderick MacKinnon[1, *].

[1]Laboratory of Molecular Neurobiology and Biophysics, Howard Hughes Medical Institute, The Rockefeller University, New York, United States.

*Correspondence to: Roderick MacKinnon.

Email: mackinn@rockefeller.edu.

This PDF file includes:

Figures S1 to S8
Table S1
Legends for Movies S1 and S2

Other supporting materials for this manuscript include the following:

Movies S1 and S2

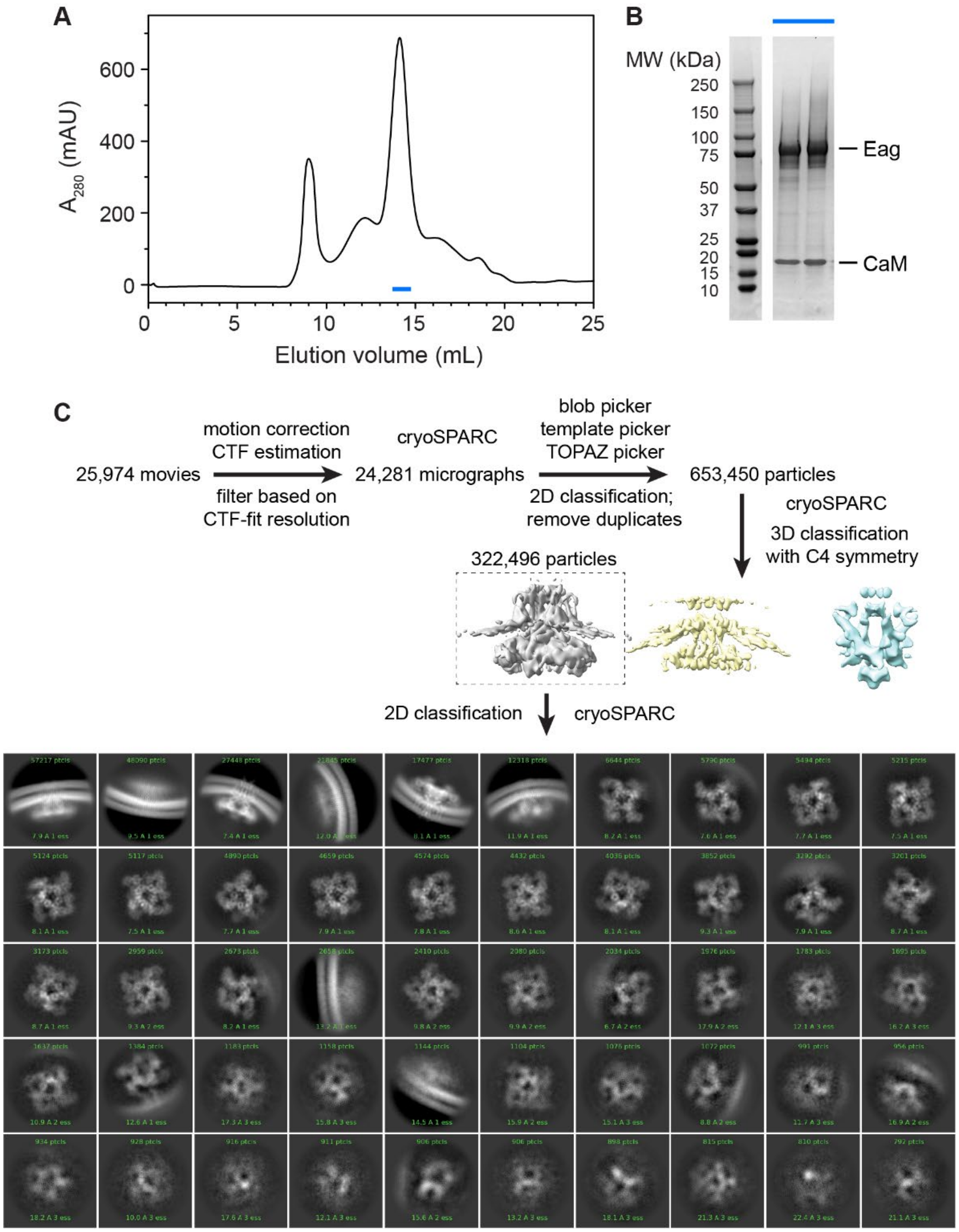

Figure S1. Purification of the rEag1-CaM complex and two-dimensional class averages of particles used for further classification.

(**A**) Gel-filtration chromatogram of the rEag1-CaM complex on a Superose 6 Increase column. The fractions labeled with a blue line (14-15 mL) were used for reconstitution. (**B**) SDS-PAGE gel of the selected fractions showing the presence of Eag and CaM. (**C**) Two-dimensional class averages for a subset of particles used for 3D classification are shown. The classes were sorted by their populations and the top 40 classes (out of 100) are shown. All side views show protein density inside the vesicle but not outside the vesicle, indicating that channels are inserted only in inside-in orientations.

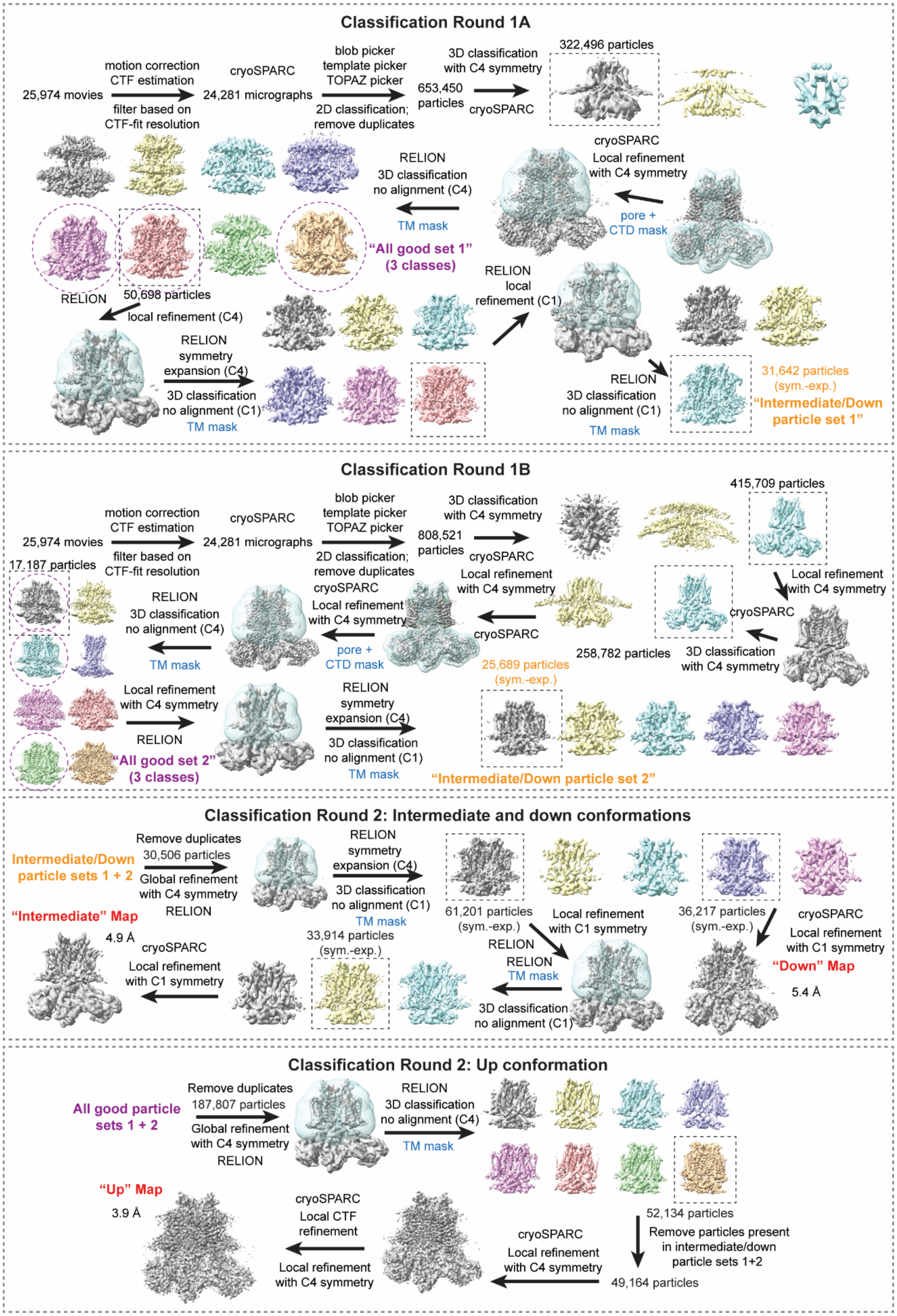
Classification Round 1A
25,974 movies
motion correction
CTF estimation
filter based on
CTF-fit resolution
cryoSPARC
24,281 micrographs
blob picker
template picker
TOPAZ picker
2D classification;
remove duplicates
653,450
particles
3D classification
with C4 symmetry
cryoSPARC
322,496 particles
cryoSPARC
Local refinement
with C4 symmetry
pore +
CTD mask
RELION
3D classification
no alignment (C4)
TM mask
"All good set 1"
(3 classes)
RELION
50,698 particles
local refinement (C4)
RELION
symmetry
expansion (C4)
3D classification
no alignment (C1)
TM mask
RELION
local
refinement (C1)
RELION
3D classification
no alignment (C1)
TM mask
31,642 particles
(sym.-exp.)
"Intermediate/Down
particle set 1"
Classification Round 1B
25,974 movies
motion correction
CTF estimation
filter based on
CTF-fit resolution
cryoSPARC
24,281 micrographs
blob picker
template picker
TOPAZ picker
2D classification;
remove duplicates
808,521
particles
3D classification
with C4 symmetry
cryoSPARC
415,709 particles
Local refinement
with C4 symmetry
cryoSPARC
3D classification
with C4 symmetry
258,782 particles
Local refinement
with C4 symmetry
cryoSPARC
cryoSPARC
Local refinement
with C4 symmetry
pore +
CTD mask
RELION
3D classification
no alignment (C4)
TM mask
17,187 particles
Local refinement
with C4 symmetry
RELION
"All good set 2"
(3 classes)
RELION
symmetry
expansion (C4)
3D classification
no alignment (C1)
TM mask
25,689 particles
(sym.-exp.)
"Intermediate/Down particle set 2"
Classification Round 2: Intermediate and down conformations
Intermediate/Down
particle sets 1 + 2
Remove duplicates
30,506 particles
Global refinement
with C4 symmetry
RELION
RELION
symmetry
expansion (C4)
3D classification
no alignment (C1)
TM mask
61,201 particles
(sym.-exp.)
Local refinement
with C1 symmetry
RELION
36,217 particles
(sym.-exp.)
cryoSPARC
Local refinement
with C1 symmetry
"Down" Map
5.4 Å
RELION
TM mask
3D classification
no alignment (C1)
33,914 particles
(sym.-exp.)
cryoSPARC
Local refinement
with C1 symmetry
"Intermediate" Map
4.9 Å
Classification Round 2: Up conformation
All good particle
sets 1 + 2
Remove duplicates
187,807 particles
Global refinement
with C4 symmetry
RELION
RELION
3D classification
no alignment (C4)
TM mask
52,134 particles
Remove particles present
in intermediate/down
particle sets 1+2
49,164 particles
cryoSPARC
Local refinement
with C4 symmetry
cryoSPARC
Local CTF
refinement
Local refinement
with C4 symmetry
"Up" Map
3.9 Å

Figure S2. Workflow for three-dimensional cryo-EM data processing.

Cryo-EM maps from refinement or classification are shown in solid surface and masks in transparent blue surface. Classes selected for further refinement are marked by a dashed grey box. The symmetry used for refinement and classification, particle counts for the selected classes and the software package used is indicated for each step.

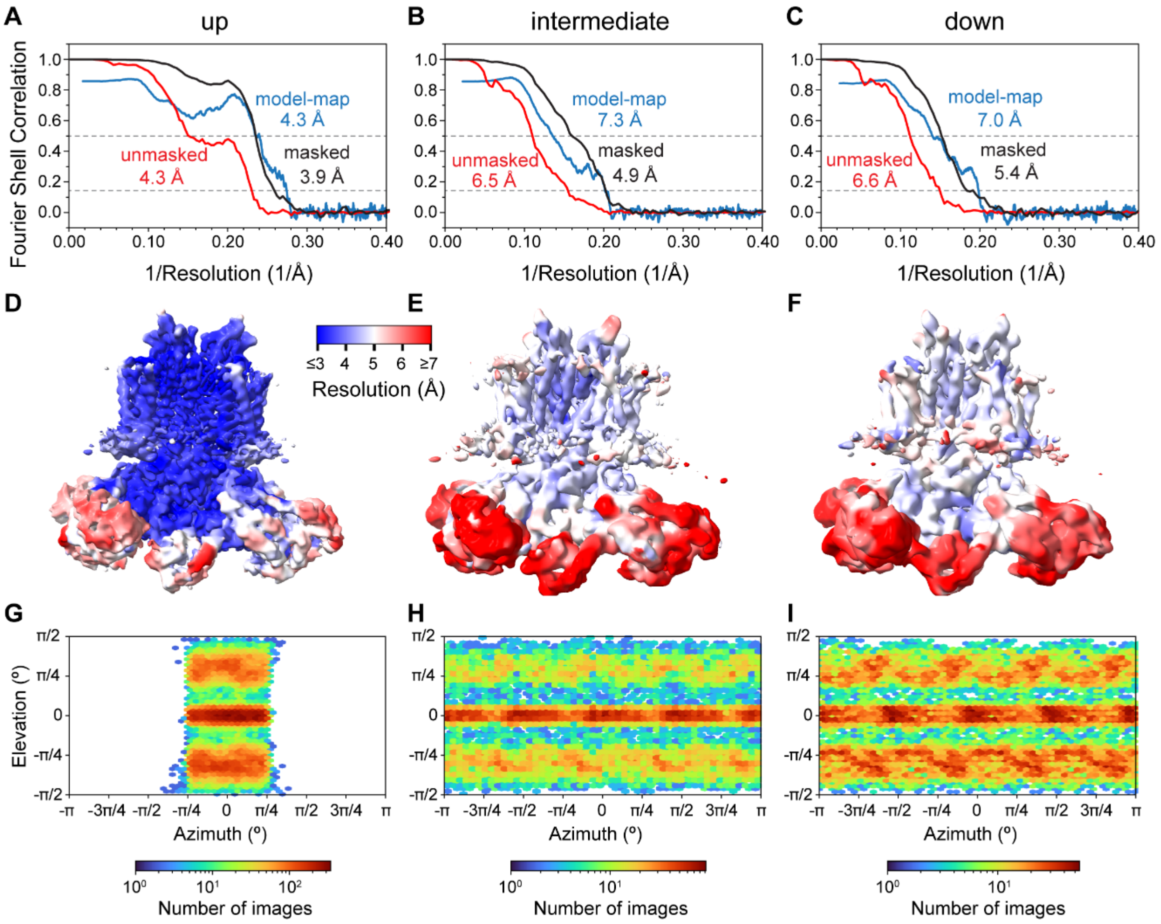

Figure S3. Fourier shell correlation curves and local resolution estimates for the cryo-EM maps.

(**A-C**) Fourier Shell Correlation (FSC) curves for the (**A**) up, (**B**) intermediate and (**C**) down maps calculated using the two independent half-maps from refinement, overlaid with the FSC curve calculated between each map and structural model (blue). FSC curves for masked maps are shown in black and those for unmasked maps are in red. The nominal resolution at the gold-standard criterion (FSC=0.143) is given for each map. (**D-F**) Local resolution estimates (FSC = 0.143) for the (**D**) up, (**E**) intermediate and (**F**) down maps overlaid with the corresponding maps. Blue regions are the highest-resolution parts (≤3 Å) of the maps and red regions the lowest-resolution parts (≥7 Å). These calculations were carried out in cryoSPARC. (**G-I)** Distribution of orientations over the elevation and azimuth angles for particles included in the calculation of the final (**G**) up, (**H**) intermediate and (**I**) down maps. The key for the heat map is included below each plot.

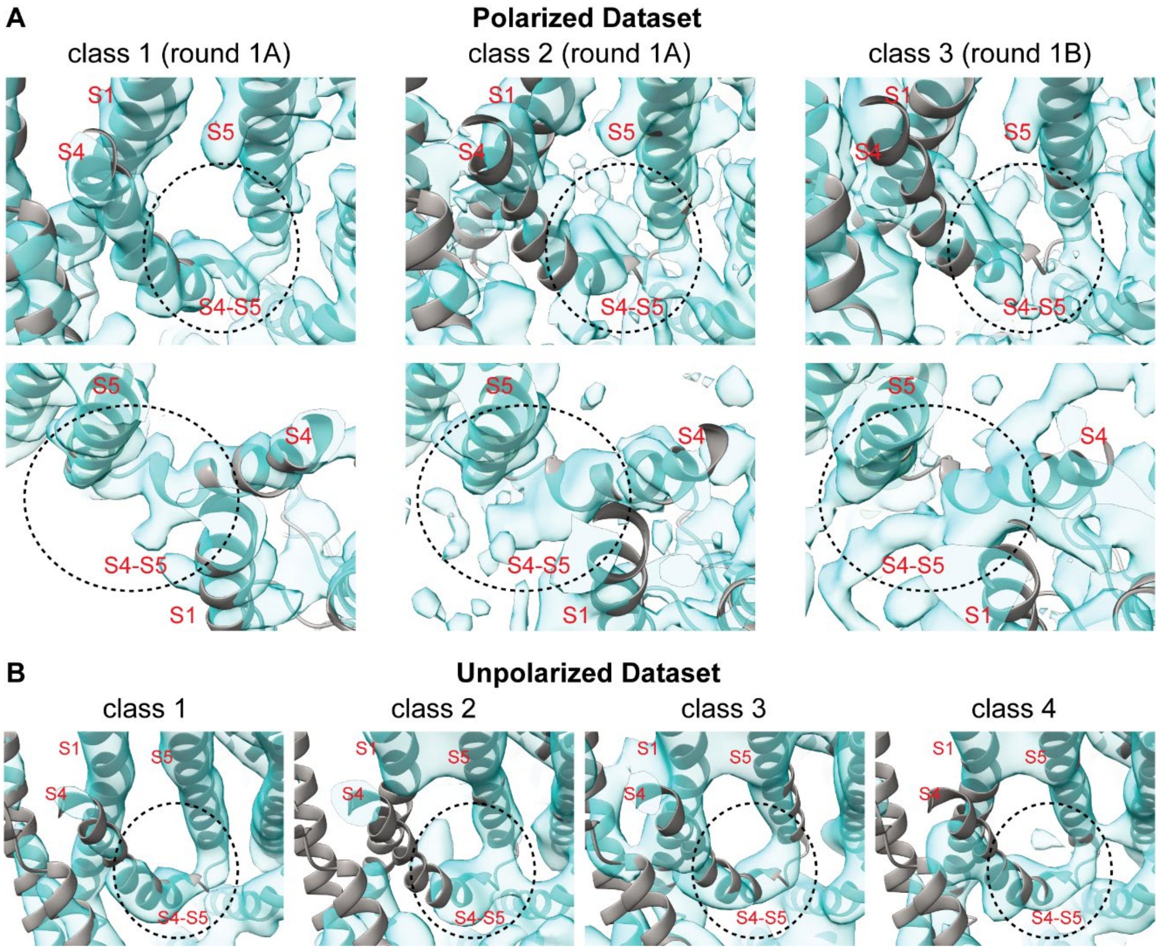

Figure S4. 3D classification of Eag in polarized and unpolarized vesicles.

(**A**) Three classes (left to right) from the polarized dataset with different voltage sensor conformations, showing two different views (top and bottom). The cryo-EM density (translucent blue surface) for each class is overlaid with the up conformation shown as grey cartoons. The densities for classes 2 and 3 do not agree with the up conformation: the S4-S5 linker extends in the direction into the page instead of connecting to the bottom of S5 as in the model. Subsets of the particles in classes 2 and 3 were used to obtain the intermediate and down maps, while part of those in class 1 were used to calculate the up map. (**B**) Cryo-EM density for the four classes in the unpolarized vesicle dataset with visible voltage sensor density, overlaid with the up conformation as above. All classes in the unpolarized dataset are consistent with the up conformation.

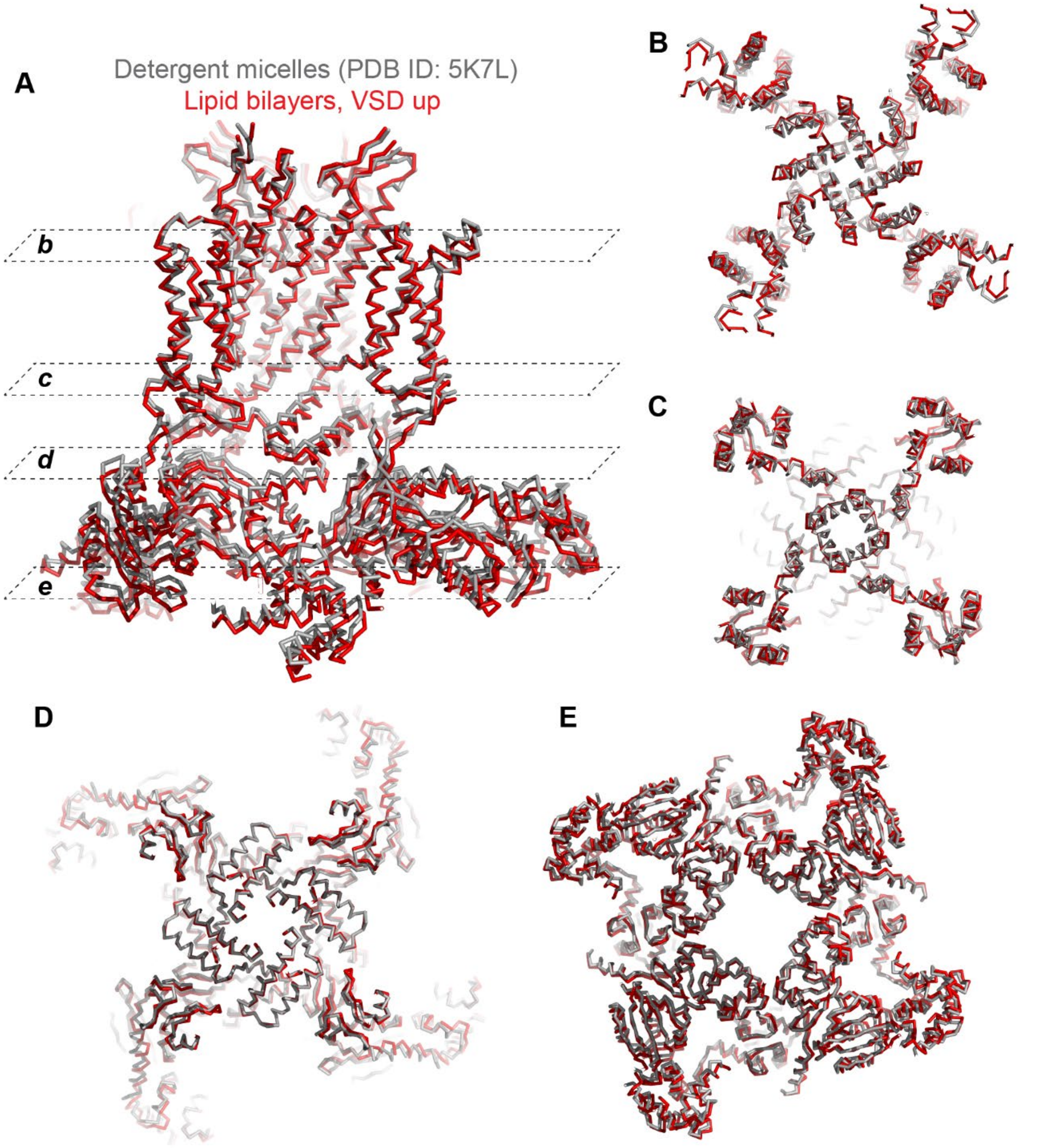

Figure S5. Comparison of Eag structure in lipid bilayers and detergent micelles.

(**A**) Side view of the Eag up structure solved here in lipid bilayers (red) and that previously determined in detergent micelles (grey, PDB ID: 5K7L) (18). The structures are aligned by their selectivity filters and pore helices. Both structures are very similar. The cytoplasmic domain shows a slight downward displacement and counterclockwise rotation (from the extracellular side), and the transmembrane domain is slightly (~0.5 Å) expanded radially in lipid bilayers. (**B-E**) Top-down views (from the extracellular side) of the channel at various slices indicated in (A). (**B**) At the top of the transmembrane domain. (**C**) Towards the bottom of the transmembrane domain. (**D**) Near the C-linker. (**E**) In the cytoplasmic domain.

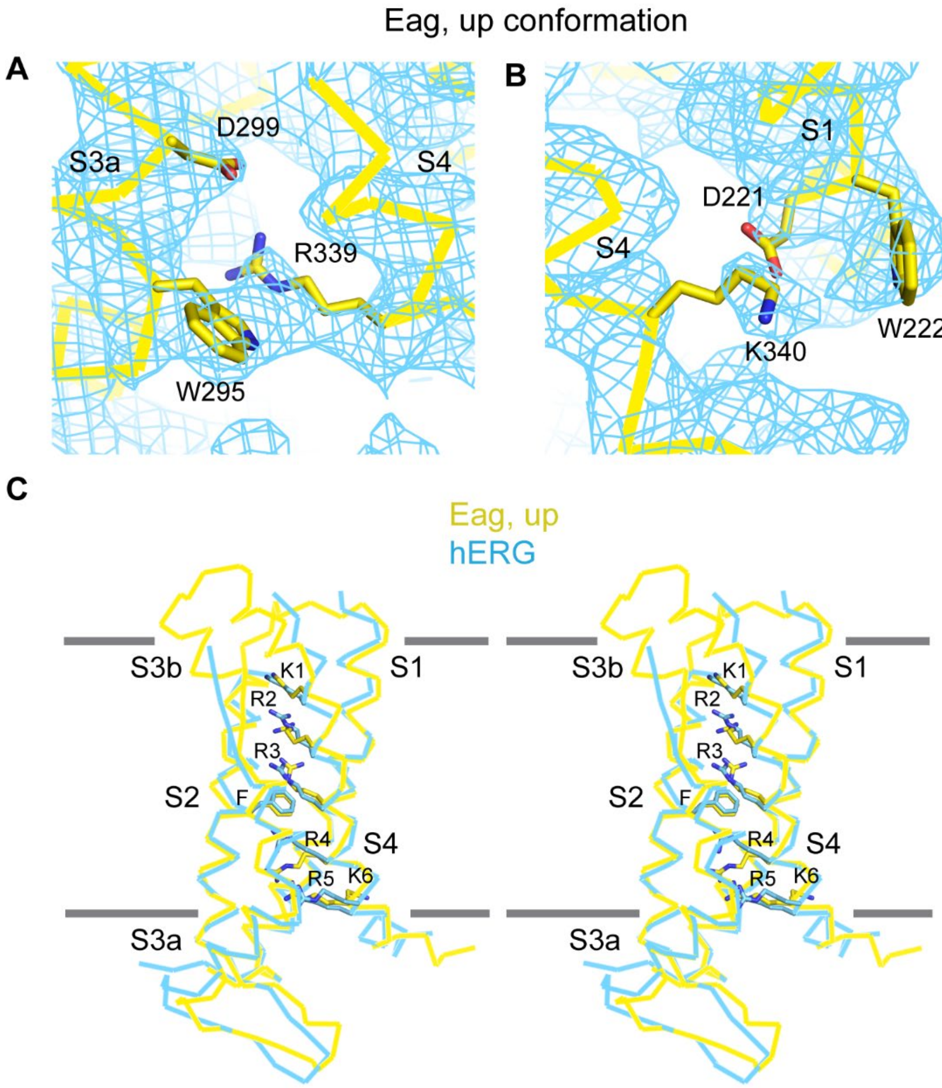

Figure S6. Additional views of Eag in the up conformation and comparison of voltage sensor domains in Eag and hERG.

(**A, B**) Residues close to the bottom two positive charges from S4 in the up structure. The channel is shown in Cα trace representation with sticks for the residues of interest and cryo-EM density from the up map is overlaid as blue mesh. (**A**) R5 (R339) is close to D299 and W295 in S3. (**B**) K6 (K340) is close to D221 and W222 in S1. (**C**) Stereoview of voltage sensor domains in Eag (yellow) with the pore closed and S4 up and hERG (blue) with the pore open and S4 up. The structures are shown in Cα trace representation and sticks are shown for the six positively charged residues and the Phe in the gating charge transfer center. The voltage sensor structures are very similar in the up conformation.

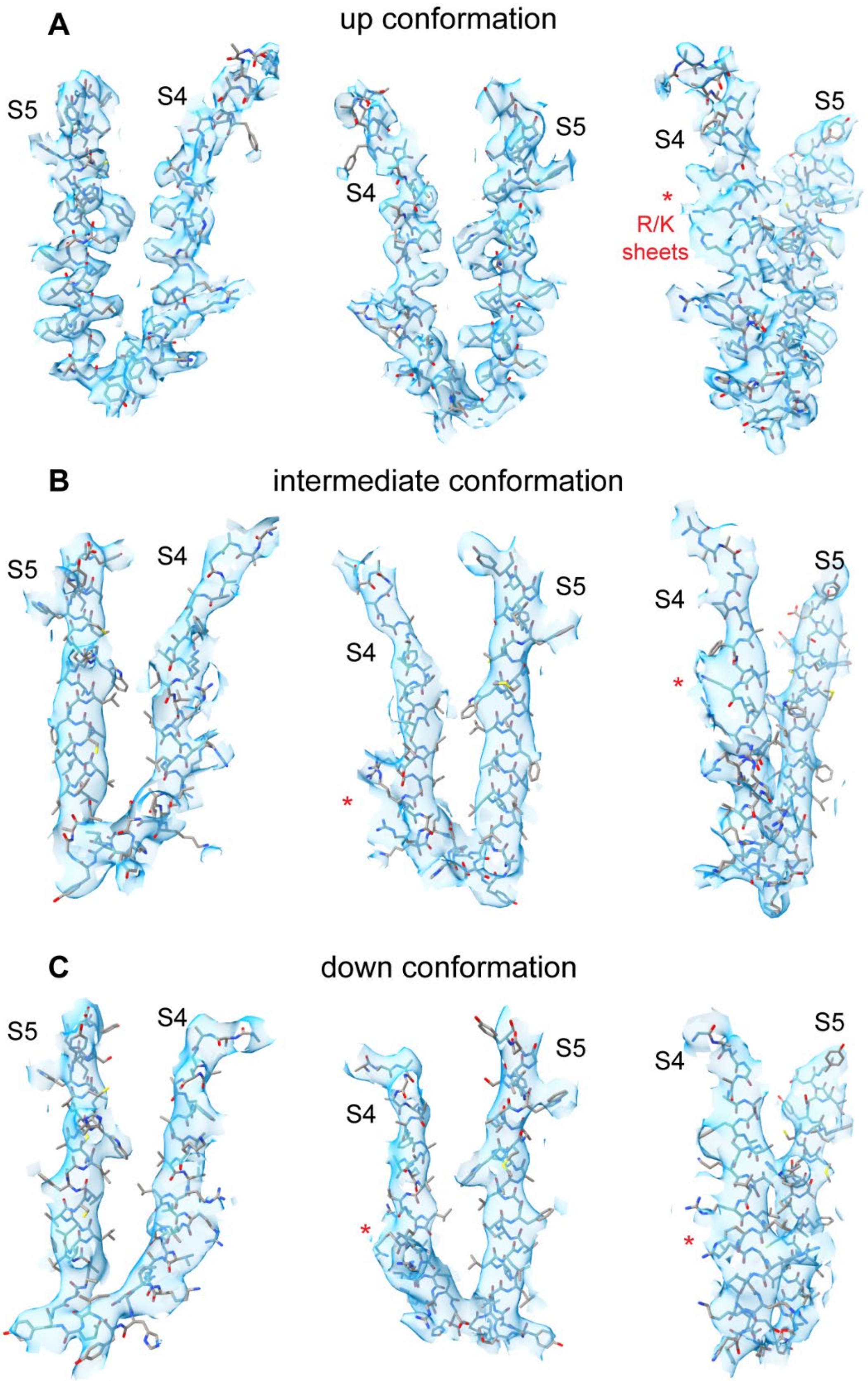

A
up conformation
S5
S4
S4
S5
S4
S5
*
R/K
sheets
B
intermediate conformation
S5
S4
S4
S5
*
S4
S5
*
C
down conformation
S5
S4
S4
S5
*
S4
S5
*

Figure S7. Cryo-EM densities for S4 to S5 in the three conformations.

(**A-C**) Three different views of cryo-EM density and structural models for residues 315-376 (including S4 and S5) for the (**A**) up, (**B**) intermediate and (**C**) down maps. The locations of continuous arginine and lysine density in the three maps are marked by red asterisks. The maps are shown as transparent blue surfaces and the models are shown in stick representation.

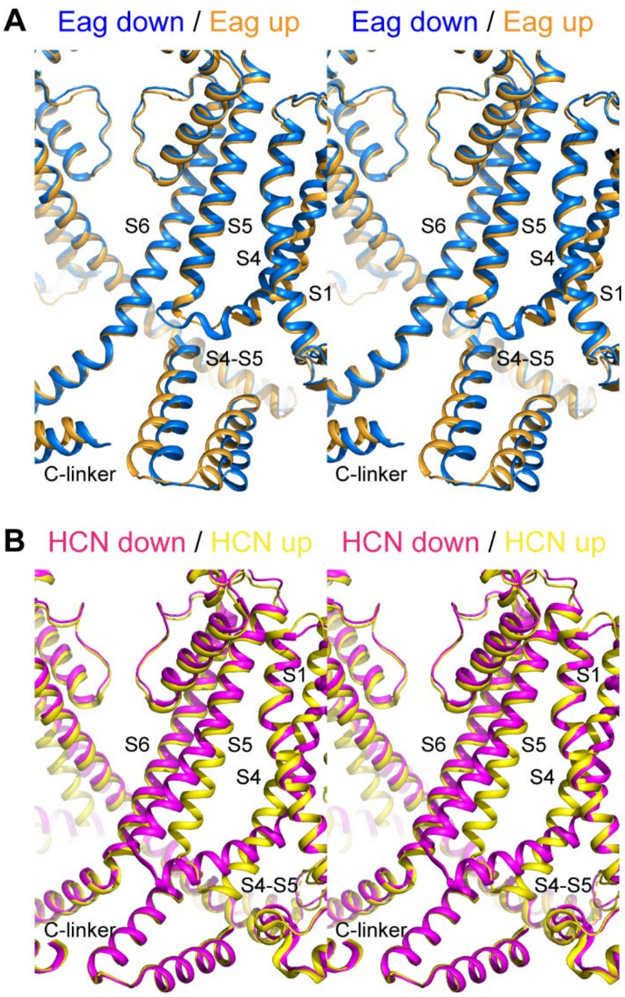

Figure S8. Coupling between the voltage sensor and the pore in depolarization- and hyperpolarization-activated channels.

(**A**) Stereoview of the depolarization-activated Eag with pore closed and S4 down (blue) and with pore closed and S4 up (orange). (**B**) Stereoview of the hyperpolarization-activated HCN with pore closed and S4 up (yellow) (32) and with pore in a pre-open conformation and S4 down (magenta) (10). The transmembrane domain and C-linker of the subunit with structural elements labeled, the transmembrane domain of the diagonally opposite subunit, and the C-linker of the adjacent subunit are shown in cartoon representation.

Table S1. Summary of cryo-EM reconstruction and structural model statistics.

Reconstructions	Up	Intermediate	Down
Microscope/Camera		Titan Krios 2 300 kV / Gatan K3	
Pixel Size		1.08 Å	
Total dose		56 e⁻/Å^2	
Defocus range		-1.0 to -2.0 μm	
Movies collected		25,794	
Particle number	49,164	33,914	36,217
Symmetry imposed	C4	C1	C1
Overall resolution (masked)	3.9 Å	4.9 Å	5.4 Å
**Models**	**Up**	**Intermediate**	**Down**
**Ramachandran plot**			
Preferred (%)	95.96	96.41	96.53
Allowed (%)	4.04	3.59	3.47
Outliers (%)	0.00	0.00	0.00
**MolProbity**			
Clash Score	8.15	12.46	13.59
Rotamer Outliers (%)	0.35	0.00	0.00
Cβ deviations	0	0	0
Overall Score	1.72	1.85	1.87
**RMS deviations**			
Bond lengths (Å)	0.003	0.004	0.003
Bond angles (°)	0.573	0.867	0.731

Movie S1 (separate file). Sequence of conformational changes occurring during channel gating.

The movie is a morph between three structures: Eag with the pore closed and VSD down, Eag with the pore closed and VSD up, and hERG with the pore open and VSD up. The channel is shown in cartoon representation with S4 helices (blue), S6 helices (red) and the C-linkers (green) highlighted for clarity. The movie starts with the protein in the hyperpolarized configuration, i.e. with pore closed and VSD down. To open the gate, the interfacial segment of S4 must first move out of the way. This occurs when the membrane is depolarized and the voltage sensors move up. The pore is subsequently permitted to open. This sequence is shown in reverse to show how the channel might close upon hyperpolarization. The first set of views is a side view of the channel in the membrane while the second is a top-down slice viewed from the extracellular side.

Movie S2 (separate file). Simplified view of the sequence of conformational changes occurring during channel gating.

The movie is a morph between three structures: Eag with the pore closed and VSD down, Eag with the pore closed and VSD up, and hERG with the pore open and VSD up. The channels were aligned using the selectivity filter and the pore helix. The transmembrane domain and C-linker of diametrically opposite subunits, and the C-linker of an adjacent subunit are shown in cartoon representation.
